# Adenosine monophosphate–activated protein kinase activation is associated with suppression of NLRP3 inflammasome–mediated neuroinflammation in a weight‐drop model of traumatic brain injury in Sprague–Dawley rats

**DOI:** 10.1002/ame2.70288

**Published:** 2026-09-17

**Authors:** Triveni Kodi, Paka Sravan Kumar, Krishnadas Nandakumar, Anoop Kishore

**Affiliations:** ^1^ Department of Pharmacology Manipal College of Pharmaceutical Sciences, Manipal Academy of Higher Education Manipal India

**Keywords:** adenosine monophosphate–activated protein kinase (AMPK), neuroinflammation, nucleotide‐binding oligomerization domain, leucine‐rich repeat, and pyrin domain‐containing protein 3 (NLRP3), traumatic brain injury, weight‐drop method

## Abstract

**Background:**

Traumatic brain injury (TBI) disrupts adenosine monophosphate–activated protein kinase (AMPK) signaling, impairs energy homeostasis, activates inflammatory pathways, and triggers neuroinflammation. AMPK activators such as metformin (MET), quercetin (QUE), resveratrol (RES), cinnamaldehyde (CIN), and berberine (BER) exhibit anti‐inflammatory and neuroprotective properties. However, their role in modulating the nucleotide‐binding oligomerization domain, leucine‐rich repeat, and pyrin domain‐containing protein 3 (NLRP3) inflammasome in TBI‐induced neuroinflammation remains unclear. This study investigated the neuroprotective effects of AMPK activators in the weight‐drop model of TBI, focusing on suppression of the NLRP3 inflammasome.

**Methods:**

Rats received an intraperitoneal administration of AMPK activators—MET (100 mg/kg), QUE (15 mg/kg), RES (25 mg/kg), CIN (25 mg/kg), or BER (50 mg/kg)—from days −3 to 17. TBI was induced on day 1 using the weight‐drop method (240 g from 1.5 m). Cognitive performance was evaluated using the novel object recognition test and Morris water maze. Neuroinflammatory markers, including nuclear factor kappa B (NF‐κB), NLRP3, pro‐caspase‐1, interleukin‐1β (IL‐1β), tumor necrosis factor‐α (TNF‐α), and microglial polarization markers (CD86 and CD206), were assessed using Western blotting and enzyme‐linked immunosorbent assay (ELISA). Oxidative stress and histopathological changes were also examined.

**Results:**

TBI resulted in cognitive impairment, oxidative stress, and neuronal damage; reduced p‐AMPK/AMPK ratio; decreased CD206 levels; and elevated the levels of NF‐κB, CD86, TNF‐α, and NLRP3 inflammasome components (NLRP3, pro‐caspase‐1, and IL‐1β). Pretreatment with AMPK activators significantly improved cognitive deficits, attenuated oxidative stress and neuroinflammation, restored p‐AMPK/AMPK ratio, promoted anti‐inflammatory effects, and suppressed NLRP3 inflammasome activation.

**Conclusion:**

AMPK activators exhibited neuroprotection in TBI by improving cognition and regulating oxidative stress, microglial activation, and NLRP3‐mediated neuroinflammation.

## Graphical abstract

This graphical abstract illustrates the study's schematic model. Adenosine monophosphate–activated protein kinase (AMPK) activators exhibited neuroprotection in the weight‐drop model of traumatic brain injury by improving cognition and regulating oxidative stress, microglial activation, and nucleotide‐binding oligomerization domain, leucine‐rich repeat, and pyrin domain‐containing protein 3 (NLRP3)‐mediated neuroinflammation.

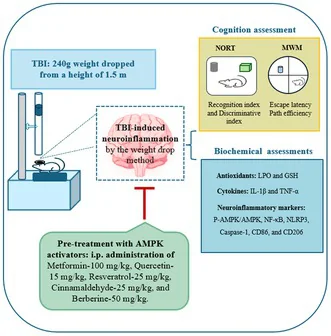

## 1 INTRODUCTION

Traumatic brain injury (TBI) is a complex condition involving both primary and secondary injury mechanisms that lead to structural damage and functional deficits. Primary injury occurs immediately upon exposure to external force, causing contusion, hemorrhage, and axonal shearing. In contrast, secondary injury evolves over hours to months due to cascades of metabolic, cellular, and molecular events, ultimately leading to brain cell death, tissue damage, and atrophy.^1^ TBI mimics brain edema and diffuse axonal damage. Both conditions are linked to disruption of the blood–brain barrier (BBB), which initiates neuroinflammatory events and inflammasome activation. Traumatic injury is followed by neuroinflammation and microglial activation, which play a crucial role in driving the pathological processes, further exacerbating brain damage.^2^, ^3^

Multiple studies have suggested that the inflammasome complex plays a key role in initiating the immune response after TBI.^4^, ^5^ In TBI, nucleotide‐binding oligomerization domain, leucine‐rich repeat, and pyrin domain‐containing protein 3 (NLRP3) inflammasome expression increases significantly, and the levels of cerebrospinal fluid (CSF) fluid peak on day 1, decrease on day 2, and then increase again on days 3 and 4.^6^ Cell necrosis may cause an early spike; however, infection, cell stress, or inflammatory activity may cause later elevations. Also, NLRP3 messenger RNA (mRNA) levels peak 6 h post‐TBI, decline at 24 h, and progressively increase until day 7. This early increase may result from cytoplasmic K^+^ and ATP release from dying cells, whereas the later increase is linked to inflammation, oxidative stress, and secondary brain injury. In brain injury models, studies demonstrate that NLRP3 suppression improves neurological function and lowers inflammation,^7^, ^8^, ^9^ and reduces astrocyte and microglial activation.^5^, ^10^

Reduced adenosine monophosphate–activated protein kinase (AMPK) activation after TBI leads to a cellular energy crisis in an injured brain, resulting in memory impairment. However, in TBI rats, AMPK agonists can promote neuroprotection and enhance memory.^11^ AMPK might assist injured neurons in restoring their energy and alleviating secondary brain damage caused by TBI. Enhanced p‐AMPK/AMPK ratio mitigates the TBI‐mediated secondary injury and improves neurobehavioral function, suggesting that AMPK is required for neuroprotective effects.^1^ AMPK promotes the anti‐inflammatory (CD206) polarization marker and mitigates TBI by suppressing nuclear factor kappa B (NF‐κB).^3^

However, the neuroprotective effects of AMPK activators such as metformin (MET), quercetin (QUE), resveratrol (RES), cinnamaldehyde (CIN), and berberine (BER) in TBI, as well as the role of AMPK activation in suppressing NLRP3 inflammasome signaling pathway in TBI‐induced neuroinflammation, remain unclear. In this study, we conducted the in vivo experiments to investigate the potential neuroprotective effects of AMPK activators on TBI and further explored their role in modulating the NLRP3 inflammasome signaling pathway.

## 2 MATERIALS AND METHODS

### 2.1 Drugs and chemicals

Rat interleukin‐1β (IL‐1β), tumor necrosis factor‐α (TNF‐α), enzyme‐linked immunosorbent assay (ELISA), and primary antibodies, including p‐AMPK, AMPK, NLRP3, NF‐κB, pro‐caspase‐1, CD86, CD206, and β‐actin kits, were purchased from Elabscience Biotechnology Inc. (Houston, TX). Ketamine was procured from Neon Laboratories Ltd., and xylazine was purchased from Indian Immunologicals Limited.

MET, QUE, RES, CIN, and BER were obtained from Tokyo Chemical Industry Pvt. Ltd. (New Delhi, India). Bromophenol blue, 2‐mercaptoethanol, ammonium persulfate, sodium dodecyl sulfate (SDS), Tetramethylethylenediamine (TEMED), and Precision Plus Std Dual Color were purchased from Bio‐Rad (Hercules, CA, USA). Bovine serum albumin (BSA) and tris free base were purchased from Himedia Laboratories (Mumbai, India). Sodium chloride, Tween 20, and glycine were purchased from Sisco Research Laboratories (SRL) (Mumbai, India). Polyvinylidene fluoride (PVDF) membrane (Amersham) was purchased from Cytiva (Marlborough, MA), and bicinchoninic acid (BCA) protein assay kit was obtained from Cyanagen Srl (Bologna, Italy). Horseradish peroxidase (HRP)–conjugated goat anti‐rabbit immunoglobulin G (IgG) were purchased from Invitrogen Bio Services Pvt. Ltd. (Bangalore, India), and an enhanced chemiluminescence (ECL) substrate was procured from Takara Bio Pvt. Ltd. (New Delhi, India).

### 2.2 Experimental animals

Male Sprague–Dawley rats (10–12 weeks old) weighing 300–350 g were obtained from the Central Animal Research Facility (CARF), Manipal Academy of Higher Education, Manipal, India. All animals were housed in sterile polypropylene cages at a temperature of 23 ± 2°C and a humidity of 50% ± 5%, with a 12‐h light–dark cycle. The animals were fed a standard rodent diet in pellet form and provided water ad libitum.

The study was approved by the IAEC (no. IAEC/KMC/100/2022), Kasturba Medical College, Manipal Academy of Higher Education, Manipal, India. The animal experiments were conducted in accordance with the guidelines for the use and care of laboratory animals by the Committee for Control and Supervision of Experiments on Animals (CCSEA), Government of India.

### 2.3 Preparation and administration of drugs

MET (100 mg/kg, bodyweight b.w.), QUE (15 mg/kg, b.w.), RES (25 mg/kg, b.w.), CIN (25 mg/kg, b.w.), and BER (50 mg/kg, b.w.) were prepared as solutions in water for injection and administered intraperitoneally.

### 2.4 Weight‐drop model of TBI

**FIGURE 1:**
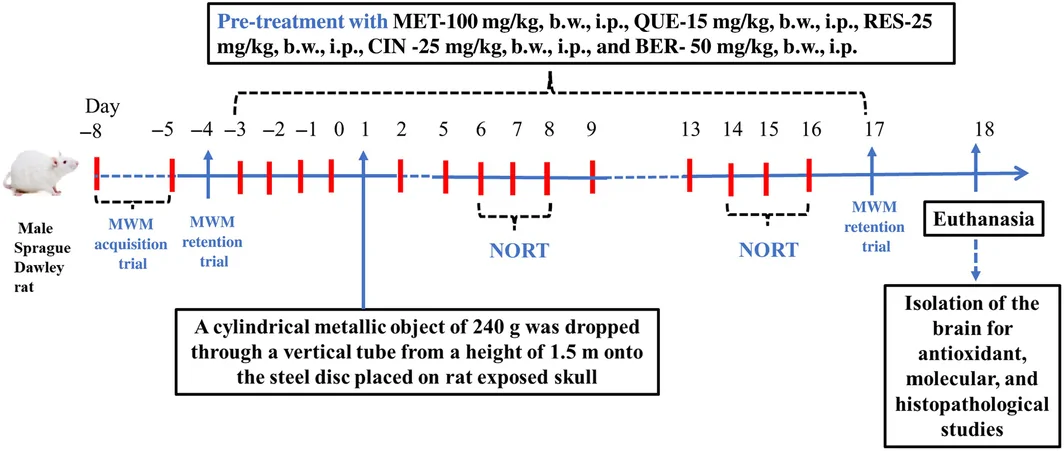
Schematic representation of the experimental methodology of TBI (traumatic brain injury)–induced neuroinflammation in rats.

TBI in Sprague–Dawley rats was induced using the weight‐drop method with minor modifications to the previously reported protocol.^12^ On day 1, animals were anesthetized using xylazine 10 mg/kg and ketamine 60 mg/kg intraperitoneally. The skull area was shaved. A 1‐ to 1.5‐cm incision was made on the skin to expose the bregma and lambda. A stainless‐steel disc (10 mm diameter and 3 mm thickness) was placed on the exposed skull to prevent skull fracture. Afterward, the rats were placed on a foam mattress to diffuse the brain injury and prevent secondary injuries. A cylindrical metallic object (240 g) was allowed to fall through a vertical tube from 1.5 m onto the steel disc. Later, the steel disc was removed, and the incision was carefully sutured^13^ (Figure 1). The topical application of povidone‐iodine solution was applied to prevent infection. In the sham group, animals underwent the same surgical procedure but without any weight drop.

### 2.5 Experimental methodology

In this study, the day of TBI induction was marked as day 1 and considered as the reference point for all subsequent procedures and treatments.

Initially, the Morris water maze (MWM) test was performed to randomly assign the animals (4 days of acquisition trial on days −8, −7, −6, and −5 and retention trial on day −4). Based on the escape latency, the animals were randomly assigned into seven groups as described here, and treatments started from day −3 and continued through day 17. On day 1, the weight‐drop method was employed to produce TBI in the rats. On day 18, the animals were sacrificed, and the whole brains were collected for antioxidant, molecular, and histopathological studies.

### 2.6 Treatment groups

The animals were divided into seven groups (Figure 1; Table 1):

- Group 1 (sham control): the animals underwent all the procedures identical to those of Group 2, except the weight‐drop impact.
- Group 2 (TBI control): a cylindrical metallic object weighing 240 g was allowed to fall from a vertical tube of 1.5 m onto the steel disc placed on the skull of the rat.
- Groups 3–7 received MET (100 mg/kg, b.w.), QUE (15 mg/kg, b.w.), RES (25 mg/kg, b.w.), CIN (25 mg/kg, b.w.), and BER (50 mg/kg, b.w.), respectively, via the intraperitoneal route in addition to weight drop.

**TABLE 1:** The treatment groups, doses, and administration.

Groups	Treatment	Dosing and administration	Number of animals
Group 1	Sham control	The animals underwent all the procedures identical to those of Group 2, except the weight‐drop impact	8
Group 2	TBI control	A cylindrical metallic object weighing 240 g was dropped from a vertical tube from a height of 1.5 m onto the steel disc placed on the rat's skull	8
Group 3	Metformin + TBI	Metformin (100 mg/kg, b.w.) was injected intraperitoneally for 4 days before the weight drop and continued for 17 days thereafter	8
Group 4	Quercetin + TBI	Quercetin (15 mg/kg, b.w.) was injected intraperitoneally for 4 days before the weight drop and continued for 17 days thereafter	8
Group 5	Resveratrol + TBI	Resveratrol (25 mg/kg, b.w.) was injected intraperitoneally for 4 days before the weight drop and continued for 17 days thereafter	8
Group 6	Cinnamaldehyde + TBI	Cinnamaldehyde (25 mg/kg, b.w.) was injected intraperitoneally for 4 days before the weight drop and continued for 17 days thereafter	8
Group 7	Berberine + TBI	Berberine (50 mg/kg, b.w.) was injected intraperitoneally for 4 days before the weight drop and continued for 17 days thereafter	8

Abbreviation: TBI, traumatic brain injury.

In Groups 3–7, treatment with the test drugs was started on day −3 and continued until day 17.

### 2.7 Behavioral tests

The MWM acquisition trial was performed on days −8, −7, −6, and −5, and the retention trial was conducted on days −4 and 17. Novel Object Recognition Test (NORT) was performed on days 8 and 16.

#### 2.7.1 MWM

After TBI, another retention trial was conducted on day 21 to assess treatment effects on spatial and learning memory.

#### 2.7.2 NORT

NORT is frequently used in research to measure a rat's novel object recognition ability. The recognition index (RI) is the proportion of time the animal spends exploring the novel object relative to the total exploration time of both objects:

$$\mathrm{RI}=\frac{\left(\text{Time spent exploring the novel object}\right)}{\left(\text{Total exploration time}\;\left[\text{Novel}+\text{Familiar objects}\right]\right)}.$$

The discriminative index (DI) measures a rat's ability to differentiate between two objects. It is calculated as the difference between the novel and familiar objects divided by the total time spent exploring both objects:

$$\mathrm{DI}=\frac{\left(\text{Time spent exploring the novel object}-\text{Time spent exploring the familiar object}\right)}{\left(\text{Total exploration time}\;\left[\text{Novel}+\text{Familiar objects}\right]\right)}.$$

### 2.8 Antioxidant assays

#### 2.8.1 Estimation of lipid peroxidation

Malondialdehyde (MDA) levels in the brain homogenate were quantified according to the procedure in Janero^14^ and Kodi et al.^15^ Briefly, brain tissue homogenates were mixed in a 1:1 ratio with a thiobarbituric acid–trichloroacetic acid–hydrochloric acid (TBA–TCA–HCl) reaction mixture. The reaction mixture was incubated at 90°C for 30 min to allow MDA present in the sample to react with TBA, resulting in the formation of a pink chromogenic complex. After incubation, the samples were cooled and centrifuged at 10 000 rpm for 10 min at 4°C. An aliquot of 100 μL of the clear supernatant was transferred to a 96‐well plate, and absorbance was measured at 532 nm using a microplate spectrophotometer. Lipid peroxidation was expressed as nanomoles of MDA per milligram of protein.

#### 2.8.2 Estimation of reduced glutathione

The amount of glutathione (GSH) in the brain homogenate was measured following the procedure described in Kodi et al.^15^ and Moron et al.^16^ Brain tissue homogenates were first mixed with 5% TCA in equal volumes to precipitate proteins and centrifuged at 4°C (4000 rpm, 10 min), and the resulting clear supernatant was carefully collected. For GSH estimation, the supernatant was combined with phosphate‐buffered saline (PBS, pH 8.0) and 5,5′‐dithiobis(2‐nitrobenzoic acid) (DTNB) in a 96‐well microplate. The plate was covered with an aluminum foil and incubated at 37°C for 10 min to allow color development. GSH reacted with DTNB to produce 5‐thio‐2‐nitrobenzoic acid (TNB), producing a yellow chromophore. Absorbance was recorded at 412 nm using a microplate reader, and GSH concentrations were calculated and expressed as micromoles per milligram of protein.

### 2.9 Western blot analysis in brain homogenate

Brain homogenates were centrifuged, and the supernatant was used for Western blot. Protein levels were measured using the BCA assay.

Primary antibodies (p‐AMPK, AMPK, NLRP3, NF‐κB, pro‐caspase‐1, CD86, CD206, β‐actin; 1:1000) and HRP‐conjugated goat anti‐rabbit IgG (1:10 000) were used. Protein bands were visualized using a chemiluminescent substrate (G Box, Chemi XRQ, Syngene, Cambridge, UK) and quantified using ImageJ software (version 1.53K, NIH, Bethesda, MD).^17^, ^18^

### 2.10 Estimation of cytokine levels in the brain

Brain IL‐1β and TNF‐α levels were quantified using rat ELISA kits (E‐EL‐R0012 and E‐EL‐R2856) as directed by the manufacturer.

### 2.11 Brain weight

After the rats were euthanized, the brains were collected and weighed to obtain the wet weight.

### 2.12 Histopathological studies in the brain

Animals were euthanized by cervical dislocation, followed by transcardial perfusion. A perfusion needle was placed into the left ventricle of the heart and gently flushed with saline, ensuring thorough clearance of blood. This was followed by perfusion with 10% neutral buffered formalin (NBF) to effectively fix the tissues. After perfusion, the brains were carefully extracted, rinsed to remove residual fixative, and postfixed in 4% formaldehyde for a minimum of 48 h. The tissues were washed, dehydrated in alcohol, and embedded in paraffin. The tissues were cut into 4‐μm sections using a microtome, floated on a water bath at 50–52°C, and mounted on slides for microscopy. Later, the sections were stained with hematoxylin and eosin (H&E).

H&E‐stained slides were used for histopathological assessment of neuronal morphology^19^, ^20^ and examined under an LX‐500 LED trinocular microscope (Labomed). Images were obtained using a MiaCam CMOS AR 6pro camera with Image AR Pro software.

### 2.13 Statistical analysis

Group comparisons were performed by employing one‐way analysis of variance (ANOVA) with Tukey's multiple comparisons test and two‐way ANOVA with Bonferroni multiple comparison test. *p* < 0.05 was considered significant.

## 3 RESULTS

### 3.1 Effects of pretreatment of AMPK activators on recognition memory using NORT in TBI‐induced neuroinflammation in rats

**FIGURE 2:**
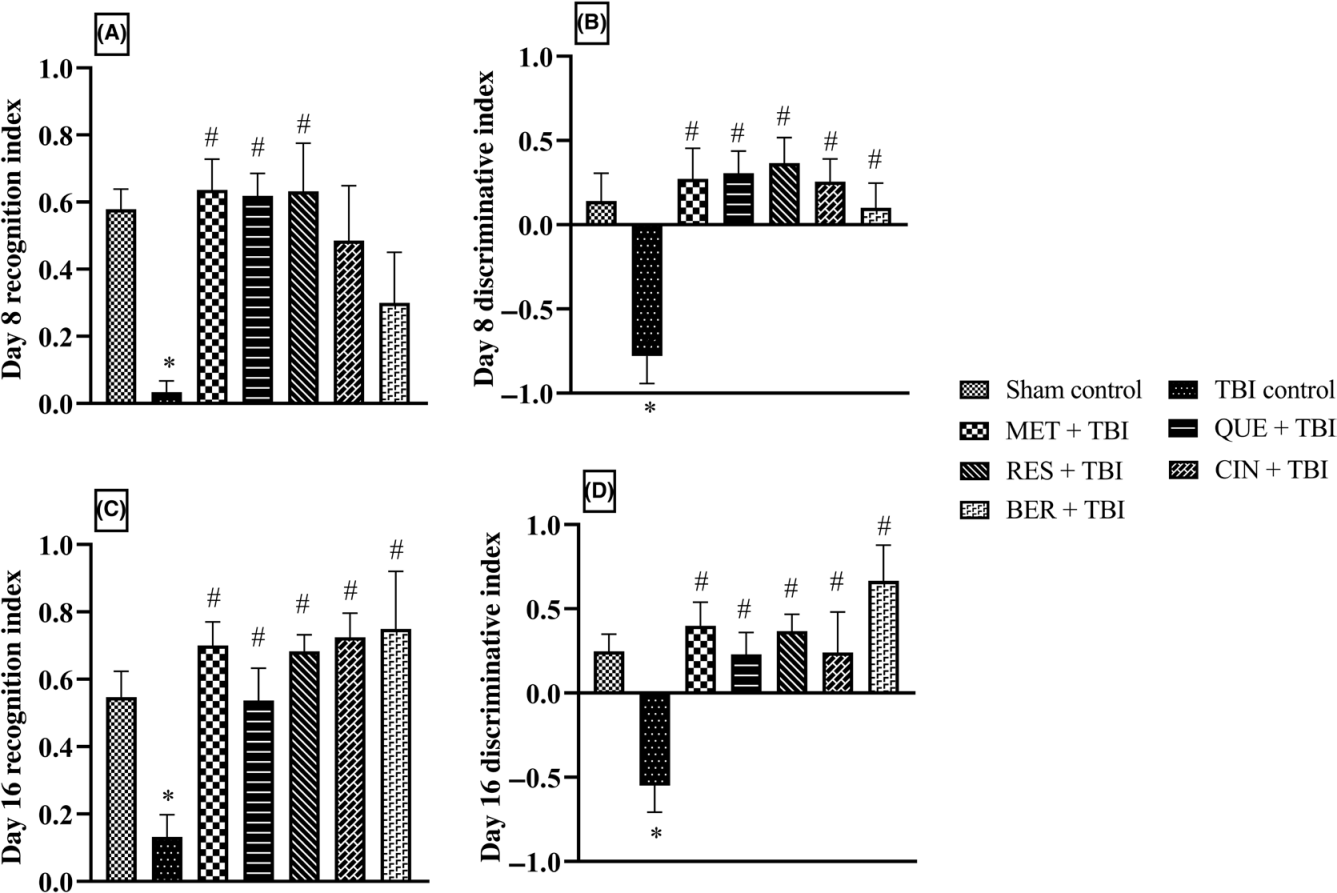
Effects of pretreatment with AMPK (adenosine monophosphate–activated protein kinase) activators on NORT: (A) recognition index (RI) on day 8, (B) discriminative index (DI) on day 8, (C) RI on day 16, and (D) DI on day 16 in TBI (traumatic brain injury)–induced neuroinflammation using weight‐drop model in rats; one‐way ANOVA (analysis of variance) and Tukey's post hoc test; mean ± SEM (standard error of the mean; *N* = 6–8). *p < 0.05 versus sham control and ^#^ *p* < 0.05 versus TBI rats. BER, berberine; CIN, cinnamaldehyde; MET, metformin; QUE, quercetin; RES, resveratrol.

It was observed that on days 8 and 16, the RI and DI significantly decreased in TBI rats compared with sham control at *p* < 0.05 (Figure 2A–D), indicating a decline in visual recognition memory. At both these time points, pretreatment with MET significantly enhanced the lower RI and DI compared to TBI rats (*p* < 0.05). It was found that pretreatments with AMPK activators significantly improved the lower RI and DI on days 8 and 16 compared with TBI rats (*p* < 0.05) (Figure 2A–D; Table 2). However, on day 8, CIN and BER did not exhibit a statistically significant effect in enhancing the RI compared to the TBI rats. These results confirm that AMPK activators could improve recognition memory impaired by TBI.

**TABLE 2:** Effect of pretreatment with AMPK activators on NORT recognition and DIs on days 8 and 16.

Serial number	Treatment	Day 8 recognition index values (mean ± SEM)	Day 8 discriminative index values (mean ± SEM)	Day 16 recognition index values (mean ± SEM)	Day 16 discriminative index values (mean ± SEM)
1	Sham control	0.58 ± 0.06	0.14 ± 0.1	0.55 ± 0.08	0.25 ± 0.1
2	TBI control	0.03 ± 0.03*	−0.78 ± 0.1*	0.13 ± 0.07*	−0.55 ± 0.1*
3	Metformin + TBI	0.64 ± 0.09^#^	0.27 ± 0.1^#^	0.7 ± 0.07^#^	0.4 ± 0.1^#^
4	Quercetin + TBI	0.62 ± 0.06^#^	0.31 ± 0.1^#^	0.54 ± 0.1^#^	0.23 ± 0.1^#^
5	Resveratrol + TBI	0.63 ± 0.1^#^	0.37 ± 0.1^#^	0.68 ± 0.05^#^	0.37 ± 0.1^#^
6	Cinnamaldehyde + TBI	0.49 ± 0.1	0.26 ± 0.1^#^	0.73 ± 0.07^#^	0.24 ± 0.2^#^
7	Berberine + TBI	0.3 ± 0.1	0.1 ± 0.1^#^	0.75 ± 0.17^#^	0.67 ± 0.2^#^

Abbreviations: AMPK, adenosine monophosphate–activated protein kinase; SEM, standard error of the mean; TBI, traumatic brain injury.

* *p* < 0.05 versus sham control.

^#^ *p* < 0.05 versus TBI rats.

**FIGURE 3:**
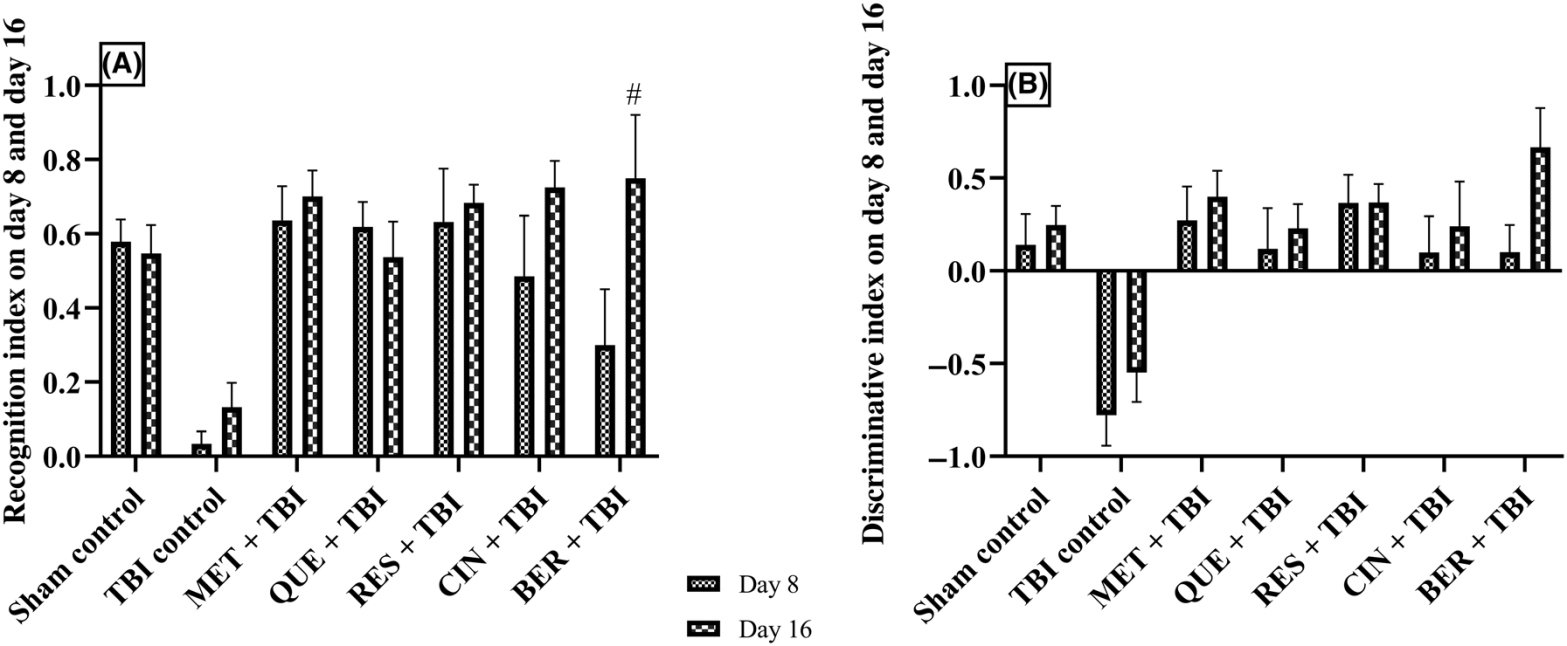
Effects of pretreatment with AMPK (adenosine monophosphate–activated protein kinase) activators on NORT: (A) recognition index (RI) on days 8 and 16, (B) discriminative index on days 8 and 16 in TBI (traumatic brain injury)–induced neuroinflammation using weight‐drop model in rats; mean ± SEM (standard error of the mean; *N* = 6–8); two‐way ANOVA (analysis of variance) followed by Bonferroni multiple comparison test. ^#^ *p* < 0.05 versus RI on day 8. BER, berberine; CIN, cinnamaldehyde; MET, metformin; QUE, quercetin; RES, resveratrol.

From days 8 to 16, there was no significant change in the RI and DI among the groups, except with BER pretreatment (Figure 3A,B).

### 3.2 Effects of pretreatment with AMPK activators on spatial learning and memory using MWM in TBI‐induced neuroinflammation in rats

**FIGURE 4:**
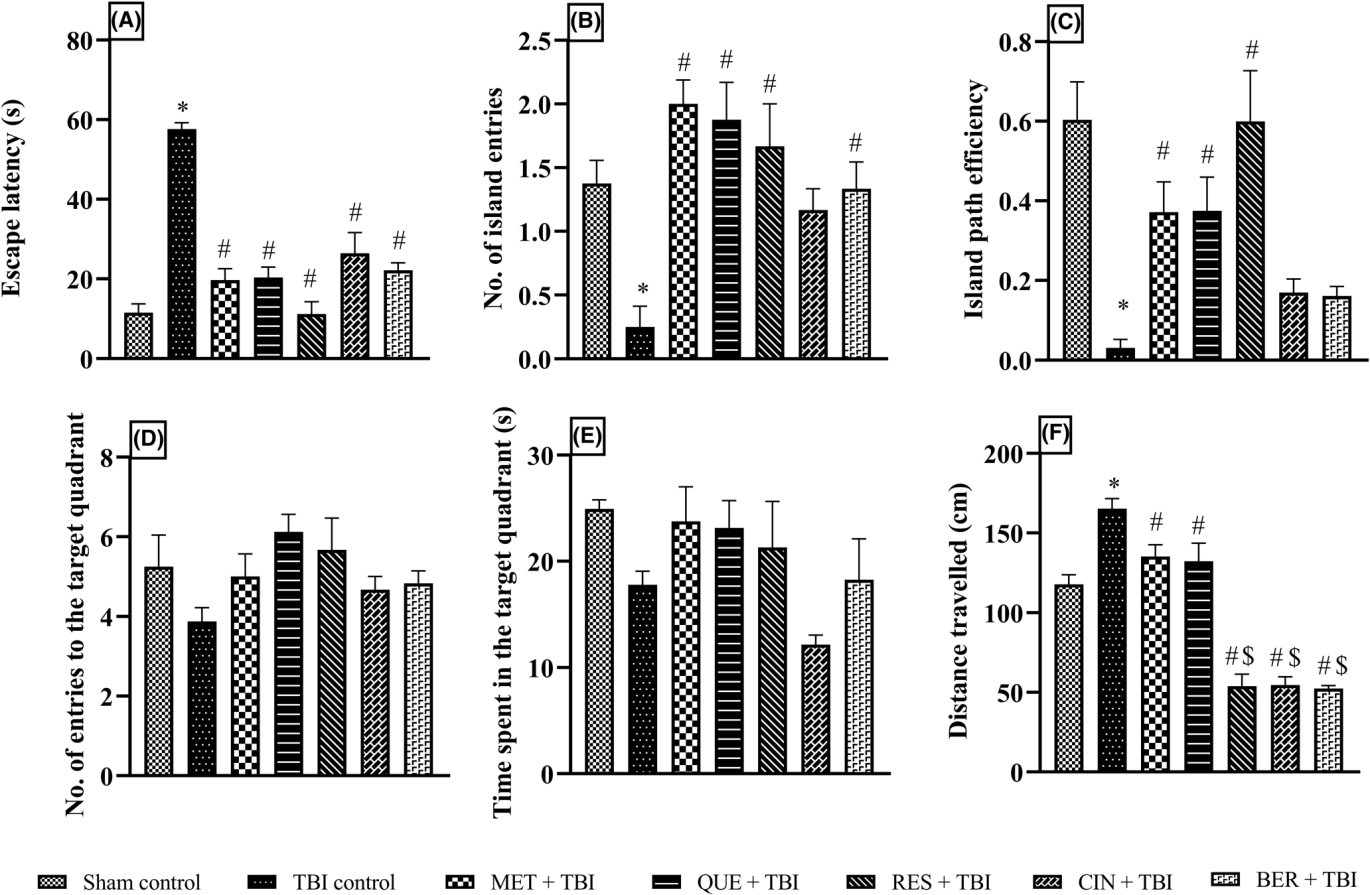
Effects of pretreatment with AMPK (adenosine monophosphate–activated protein kinase) activators on MWM (Morris water maze) spatial learning and memory: (A) escape latency, (B) number of island entries, (C) island path efficiency, (D) number of entries to the target quadrant, (E) time spent in the target quadrant, (F) distance traveled in TBI (traumatic brain injury)–induced neuroinflammation using the weight‐drop model in rats; mean ± SEM (standard error of the mean; *N* = 6–8); one‐way ANOVA (analysis of variance) and Tukey's test. *p < 0.05 versus sham control, ^#^ *p* < 0.05 versus TBI rats, and ^$^ *p* < 0.05 versus metformin pretreatment in TBI rats. BER, berberine; CIN, cinnamaldehyde; MET, metformin; QUE, quercetin; RES, resveratrol.

It was observed that with traumatic injury, the rats could not find the island in 60 s (Figure 4A–F). This was evidenced by a significant increase in escape latency and distance traveled, as well as a significant decrease in the number of island entries and island path efficiency compared with sham control (*p* < 0.05) (Table 3). Pretreatment with MET significantly attenuated the increase in escape latency and distance traveled, along with increases in the number of island entries and island path efficiency compared with the TBI rats (*p* < 0.05). There is no significant improvement in the number of entries to the target quadrant and the time spent in the target quadrant.

**TABLE 3:** Effects of pretreatment with AMPK activators on MWM spatial learning and memory in TBI‐induced neuroinflammation using weight‐drop model in rats.

Serial number	Treatment	Escape latency (mean ± SEM)	Number of island entries (mean ± SEM)	Island path efficiency (mean ± SEM)	Number of entries to the target quadrant (mean ± SEM)	Time spent in the target quadrant (mean ± SEM)	Distance traveled (mean ± SEM)
1	Sham control	11.57 ± 2.8	1.38 ± 0.1	0.6 ± 0.1	5.25 ± 0.8	24.95 ± 4.7	117.78 ± 5.9
2	TBI control	57.65 ± 1.5*	0.25 ± 0.1*	0.03 ± 0.02*	3.88 ± 0.3	17.79 ± 2.6	165.23 ± 22.8*
3	Metformin + TBI	19.68 ± 4.4^#^	2.0 ± 0.1^#^	0.37 ± 0.08^#^	5.0 ± 0.5	23.76 ± 4.3	135.27 ± 19.3^#^
4	Quercetin + TBI	20.38 ± 4.4^#^	1.88 ± 0.3^#^	0.38 ± 0.08^#^	6.13 ± 0.4	23.15 ± 2.5	132.28 ± 26.7^#^
5	Resveratrol + TBI	11.23 ± 3.0^#^	1.67 ± 0.3^#^	0.6 ± 0.1^#^	5.67 ± 0.8	21.3 ± 4.3	53.89 ± 7.6^#^, ^$^
6	Cinnamaldehyde + TBI	26.43 ± 5.1^#^	1.17 ± 0.1	0.17 ± 0.03	4.67 ± 0.3	12.17 ± 0.9	54.5 ± 5.1^#^, ^$^
7	Berberine + TBI	22.15 ± 1.8^#^	1.33 ± 0.2^#^	0.16 ± 0.02	4.83 ± 0.3	18.25 ± 3.8	52.4 ± 1.8^#^, ^$^

Abbreviations: AMPK, adenosine monophosphate–activated protein kinase; MWM, Morris water maze; SEM, standard error of the mean; TBI, traumatic brain injury.

* *p* < 0.05 versus sham control.

^#^ *p* < 0.05 versus TBI rats.

^$^ *p* < 0.05 versus metformin pretreatment in TBI rats.

Pretreatment with the AMPK activators significantly reduced escape latency and distance traveled and increased the number of island entries, except with CIN, compared with TBI rats (*p* < 0.05). Pretreatment with QUE and RES showed high island path efficiency compared with TBI rats (*p* < 0.05); however, CIN and BER did not exhibit a significant effect (Figure 4C). We found no significant change in pretreatment with AMPK activators in the number of entries to the target quadrant and the time spent in the target quadrant (Figure 4D,E). RES, CIN, and BER pretreatment significantly reduced the distance traveled compared to MET pretreatment (Figure 4F).

**FIGURE 5:**
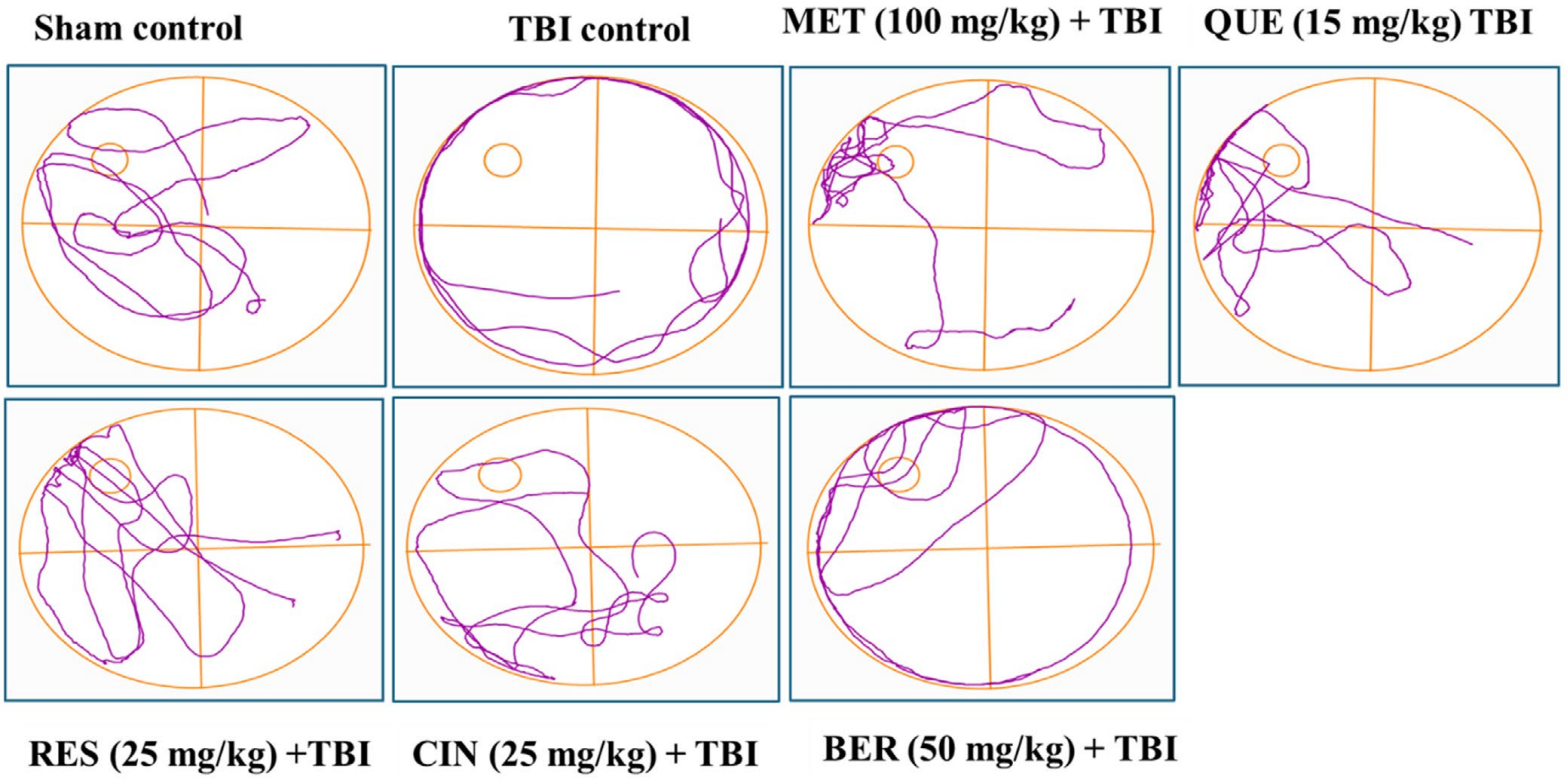
Graphical representation of the swimming track plot data from the MWM (Morris water maze) showing spatial navigation in TBI (traumatic brain injury)–induced neuroinflammation using weight‐drop model in rats with or without pretreatment using AMPK (adenosine monophosphate–activated protein kinase)–activating compounds. BER, berberine; CIN, cinnamaldehyde; MET, metformin; QUE, quercetin; RES, resveratrol.

As observed in the swimming track plots (Figure 5), TBI rats stay closer to the tank's edge and do not change their swimming direction, indicating memory impairment. Pretreatment with AMPK activators shows an inclination for movement toward the hidden platform compared with sham control. These findings confirm that AMPK activators could mitigate the spatial memory deficits caused by weight‐drop‐induced TBI.

### 3.3 Effects of pretreatment with AMPK activators on oxidative stress markers—Lipid peroxidation and reduced GSH levels—TBI‐induced neuroinflammation in rats

**FIGURE 6:**
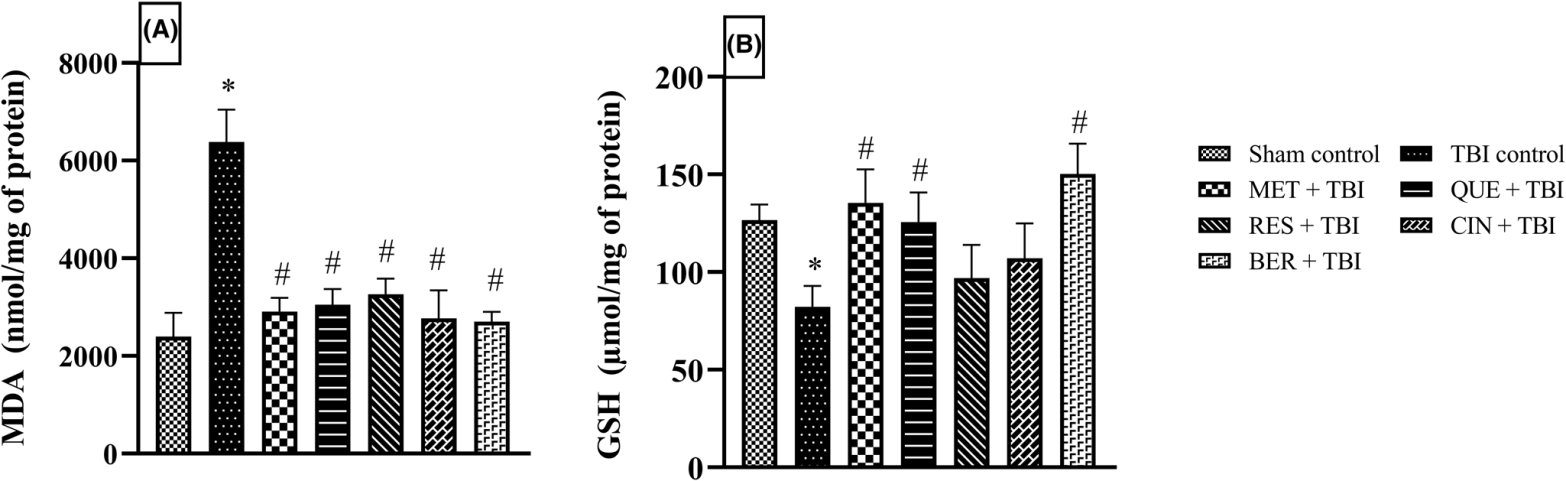
Effects of pretreatment with AMPK (adenosine monophosphate–activated protein kinase) activators on TBI (traumatic brain injury)–induced changes in rat brain homogenate: (A) MDA (malondialdehyde, nmol/mg of protein), (B) GSH (glutathione) levels (μmol/mg of protein); mean ± SEM (standard error of the mean; *N* = 3); one‐way ANOVA (analysis of variance) followed by Tukey's post hoc test. *p < 0.05 versus sham control and ^#^ *p* < 0.05 versus TBI rats. BER, berberine; CIN, cinnamaldehyde; MET, metformin; QUE, quercetin; RES, resveratrol.

Figure 6A shows a significant increase in lipid peroxidation, evidenced by elevated MDA levels in the brain homogenate supernatant in TBI rats compared with sham control (*p* < 0.05). Pretreatment with MET showed a significant reduction in MDA levels compared to TBI rats (*p* < 0.05). Similarly, pretreatment with AMPK activators exhibited a significant decrease in MDA levels compared with TBI rats (*p* < 0.05) (Table 4).

**TABLE 4:** Effects of pretreatment with AMPK activators on TBI‐induced changes in lipid peroxidation and reduced GSH levels.

Serial number	Treatment	MDA levels (mean ± SEM)	GSH levels (mean ± SEM)
1	Sham control	2396 ± 487.3	126.5 ± 3.5
2	TBI control	6382 ± 662.8*	91.2 ± 9.9*
3	Metformin + TBI	2910 ± 278.1^#^	135.4 ± 7.6^#^
4	Quercetin + TBI	3049 ± 318.8^#^	125.6 ± 7.6^#^
5	Resveratrol + TBI	3265 ± 319.0^#^	96.9 ± 8.5
6	Cinnamaldehyde + TBI	2767 ± 576.8^#^	107 ± 8.9
7	Berberine + TBI	2706 ± 193.9^#^	150.2 ± 7.7^#^

Abbreviations: AMPK, adenosine monophosphate–activated protein kinase; GSH, glutathione; MDA, malondialdehyde; SEM, standard error of the mean; TBI, traumatic brain injury.

* *p* < 0.05 versus sham control.

^#^ *p* < 0.05 versus TBI rats.

GSH levels were significantly lower in TBI rats compared to sham control (*p* < 0.05), as observed in Figure 6B. Pretreatment with MET exhibited a significant increase in GSH levels compared to TBI rats (*p* < 0.05). Pretreatment with QUE and BER significantly enhanced GSH levels compared to TBI rats (*p* < 0.05). There was an apparent, but statistically insignificant, improvement in GSH levels with RES and CIN pretreatments (Table 4).

### 3.4 Effects of pretreatment with test compounds on phosphorylated/total AMPK ratio in TBI‐induced neuroinflammation model in rats

**FIGURE 7:**
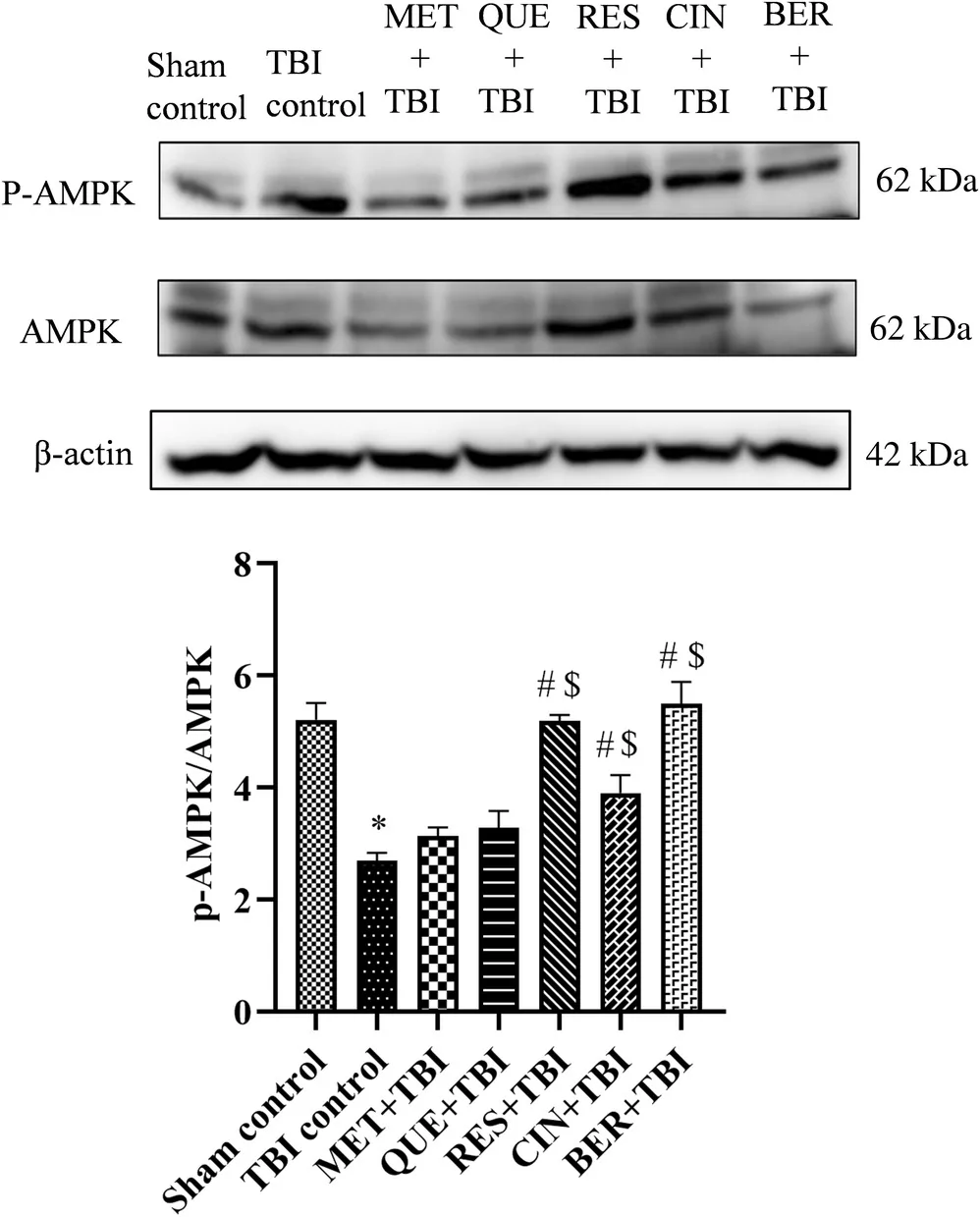
Effects of pretreatment with test compounds on phosphorylated/total AMPK (adenosine monophosphate–activated protein kinase) ratio in brain homogenates from TBI (traumatic brain injury)–induced neuroinflammation in rats were analyzed using Western blot, with β‐actin used as normalization; mean ± SEM (standard error of the mean; *N* = 3); one‐way ANOVA (analysis of variance) and Tukey's post hoc test. *p < 0.05 versus sham control, ^#^ *p* < 0.05 versus TBI rats, and ^$^ *p* < 0.05 versus metformin pretreatment in TBI rats. BER, berberine; CIN, cinnamaldehyde; MET, metformin; QUE, quercetin; RES, resveratrol.

The phosphorylated AMPK antibody detects phosphorylation at the Thr172 residue of the AMPK α subunit, whereas the total AMPK antibody recognizes total AMPK α1/2 (Thr 183/172). TBI rats exhibited a decrease in p‐AMPK/AMPK ratio compared to sham control (*p* < 0.05) (Figure 7). Pretreatment with MET and QUE showed an apparent but statistically insignificant effect in enhancing the p‐AMPK/AMPK ratio compared with TBI rats. It was found that pretreatment with RES, CIN, and BER exhibited significant enhancement in p‐AMPK/AMPK ratio compared with TBI rats and MET pretreatment (*p* < 0.05) (Figure 7; Table 5).

**TABLE 5:** Effects of pretreatment with test compounds on phosphorylated/total AMPK ratio in TBI‐induced neuroinflammation in rats.

Serial number	Treatment	p‐AMPK/AMPK levels (mean ± SEM)
1	Sham control	5.2 ± 0.1
2	TBI control	2.69 ± 0.08*
3	Metformin + TBI	3.13 ± 0.08
4	Quercetin + TBI	3.28 ± 0.1
5	Resveratrol + TBI	5.18 ± 0.06^#^, ^$^
6	Cinnamaldehyde + TBI	3.9 ± 0.1^#^, ^$^
7	Berberine + TBI	5.5 ± 0.2^#^, ^$^

Abbreviations: AMPK, adenosine monophosphate–activated protein kinase; SEM, standard error of the mean; TBI, traumatic brain injury.

* *p* < 0.05 versus sham control.

^#^ *p* < 0.05 versus TBI rats.

^$^ *p* < 0.05 versus metformin pretreatment in TBI rats.

### 3.5 Effects of pretreatment with AMPK activators on p‐NF‐κB p65 protein levels in TBI‐induced neuroinflammation in rats

**FIGURE 8:**
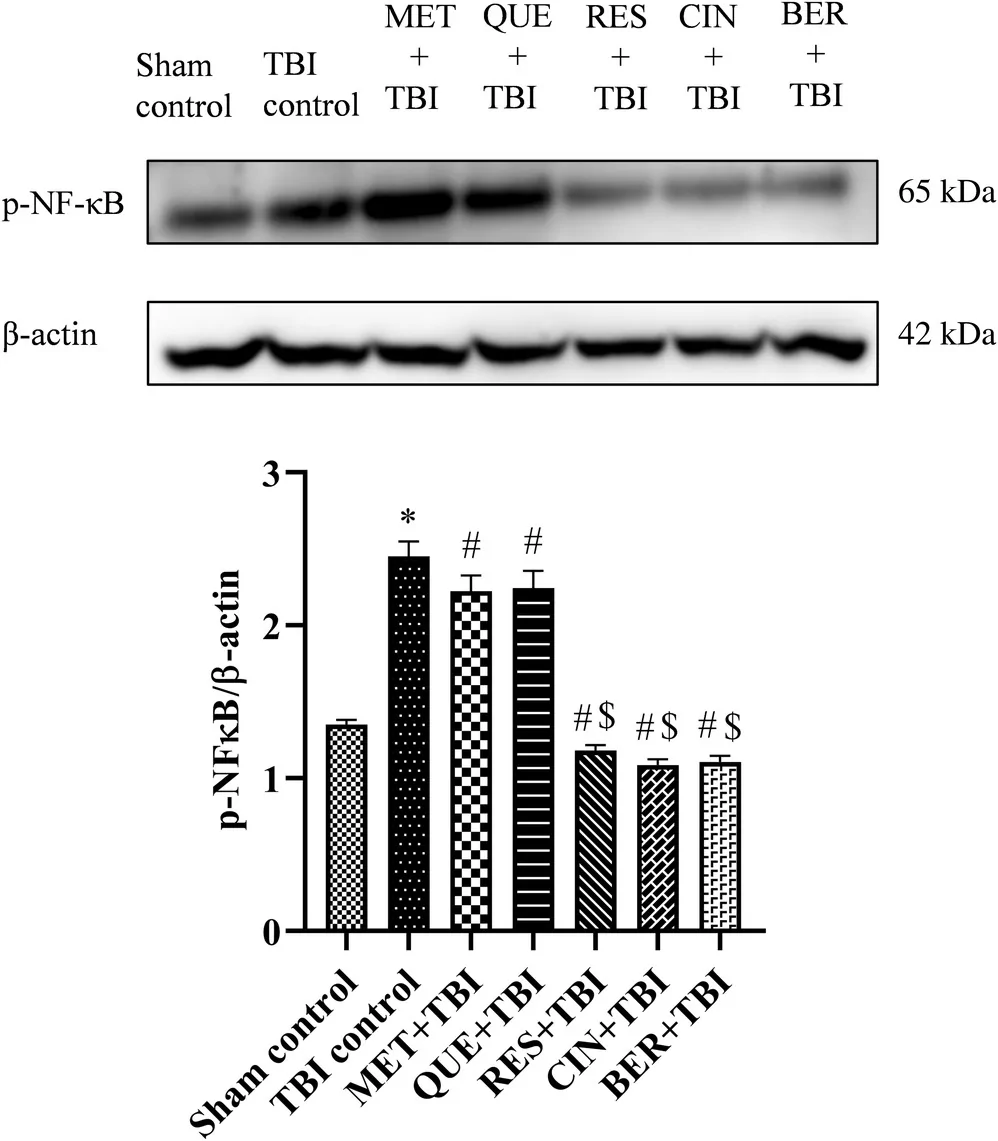
Effects of pretreatment with AMPK (adenosine monophosphate–activated protein kinase) activators on p‐NF‐κB p65 protein levels in brain homogenates from TBI (traumatic brain injury)–induced neuroinflammation in rats were analyzed using Western blot, with β‐actin used as normalization; mean ± SEM (standard error of the mean; *N* = 3); one‐way ANOVA (analysis of variance) and Tukey's post hoc test. *p < 0.05 versus sham control, ^#^ *p* < 0.05 versus TBI rats, and ^$^ *p* < 0.05 versus metformin pretreatment in TBI rats. BER, berberine; CIN, cinnamaldehyde; MET, metformin; QUE, quercetin; RES, resveratrol.

A significant increase in p‐NF‐κB p65 levels in TBI rats compared with sham control (*p* < 0.05) (Figure 8) and significantly reversed by pretreatment with MET (*p* < 0.05) was observed. Similarly, pretreatment with AMPK activators significantly reduced the TBI‐induced p‐NF‐κB p65 levels (*p* < 0.05). It was found that RES, CIN, and BER pretreatment significantly decreased TBI‐induced p‐NF‐κB p65 levels compared with MET pretreatment (Figure 8; Table 6). These findings suggest that pretreatment with AMPK activators could exhibit anti‐inflammatory effects by suppressing p‐NF‐κB in weight‐drop‐induced TBI and mitigate neuroinflammation.

**TABLE 6:** Effects of pretreatment with AMPK activators on p‐NF‐κB p65 protein levels in TBI‐induced neuroinflammation in rats.

Serial number	Treatment	p‐NF‐κB/β‐actin levels (mean ± SEM)
1	Sham control	1.35 ± 0.02
2	TBI control	2.45 ± 0.06*
3	Metformin + TBI	2.22 ± 0.06^#^
4	Quercetin + TBI	2.24 ± 0.07^#^
5	Resveratrol + TBI	1.18 ± 0.02^#^, ^$^
6	Cinnamaldehyde + TBI	1.09 ± 0.02^#^, ^$^
7	Berberine + TBI	1.11 ± 0.02^#^, ^$^

Abbreviations: AMPK, adenosine monophosphate–activated protein kinase; NF‐κB, nuclear factor kappa B; SEM, standard error of the mean; TBI, traumatic brain injury.

* *p* < 0.05 versus sham control.

^#^ *p* < 0.05 versus TBI rats.

^$^ *p* < 0.05 versus metformin pretreatment in TBI rats.

### 3.6 Effects of pretreatment with AMPK activators on NLRP3 and pro‐caspase‐1 levels in TBI‐induced neuroinflammation in rats

**FIGURE 9:**
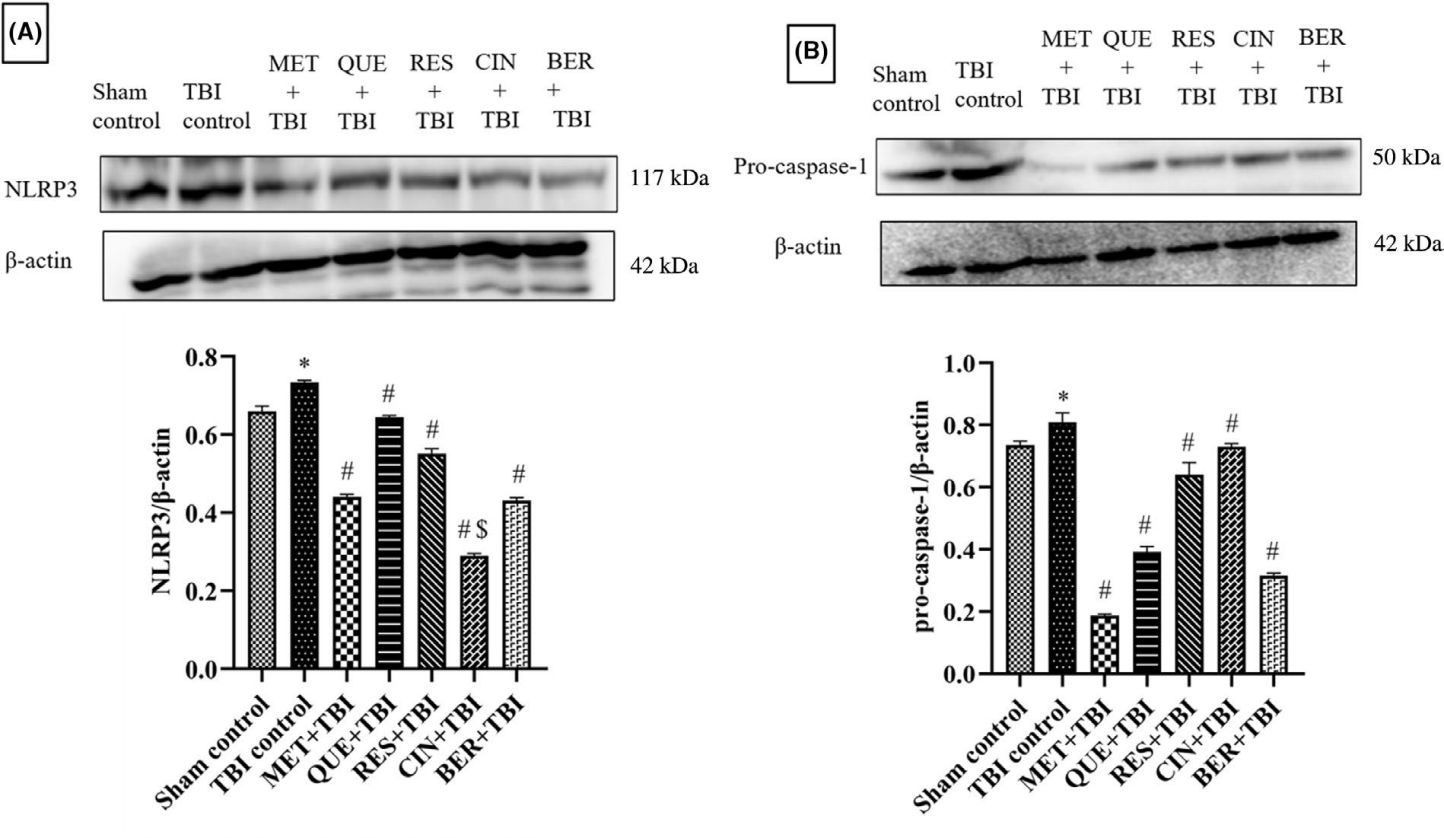
Pretreatment effects with AMPK (adenosine monophosphate–activated protein kinase) activators on (A) NLRP3 (nucleotide‐binding oligomerization domain, leucine‐rich repeat, and pyrin domain‐containing protein 3) and (B) pro‐caspase‐1 levels in brain homogenates from TBI (traumatic brain injury)–induced neuroinflammation in rats were analyzed using Western blot, with β‐actin used as normalization; one‐way ANOVA (analysis of variance) and Tukey's post hoc test; mean ± SEM (standard error of the mean; *N* = 3). *p < 0.05 versus sham control, ^#^ *p* < 0.05 versus TBI rats, and ^$^ *p* < 0.05 versus metformin pretreatment in TBI rats. BER, berberine; CIN, cinnamaldehyde; MET, metformin; QUE, quercetin; RES, resveratrol.

NLRP3 inflammasome activation, evidenced by elevated NLRP3 and pro‐caspase‐1 levels, was observed in TBI rats compared with sham control (*p* < 0.05) (Figure 9A,B). Pretreatment with MET significantly reduced the elevated levels of NLRP3 and pro‐caspase‐1 compared with TBI rats (*p* < 0.05). Similarly, pretreatment with AMPK activators showed a significant reduction in TBI‐induced NLRP3 and pro‐caspase‐1 (*p* < 0.05). Compared with MET, CIN pretreatment showed a significant change in reducing the elevated levels of NLRP3 (Figure 9A; Table 7). These results reveal that pretreatment with AMPK activators could have neuroprotective effects by inhibiting the NLRP3 inflammasome activated by weight‐drop‐induced TBI.

**TABLE 7:** Effects of pretreatment with AMPK activators on NLRP3 and pro‐caspase‐1 levels in TBI‐induced neuroinflammation in rats.

Serial number	Treatment	NLRP3/β‐actin levels (mean ± SEM)	Pro‐caspase‐1/β‐actin levels (mean ± SEM)
1	Sham control	0.66 ± 0.01	0.74 ± 0.01
2	TBI control	0.73 ± 0.002*	0.81 ± 0.02*
3	Metformin + TBI	0.44 ± 0.003^#^	0.19 ± 0.004^#^
4	Quercetin + TBI	0.64 ± 0.002^#^	0.39 ± 0.01^#^
5	Resveratrol + TBI	0.55 ± 0.01^#^	0.64 ± 0.02^#^
6	Cinnamaldehyde + TBI	0.29 ± 0.003^#^, ^$^	0.73 ± 0.01^#^
7	Berberine + TBI	0.43 ± 0.004^#^	0.32 ± 0.004^#^

Abbreviations: AMPK, adenosine monophosphate–activated protein kinase; NLRP3, nucleotide‐binding oligomerization domain, leucine‐rich repeat, and pyrin domain‐containing protein 3; SEM, standard error of the mean; TBI, traumatic brain injury.

* *p* < 0.05 versus sham control.

^#^ *p* < 0.05 versus TBI rats.

^$^ *p* < 0.05 versus metformin pretreatment in TBI rats.

### 3.7 Effects of pretreatment with AMPK activators on IL‐1β levels in TBI‐induced neuroinflammation in rats

**FIGURE 10:**
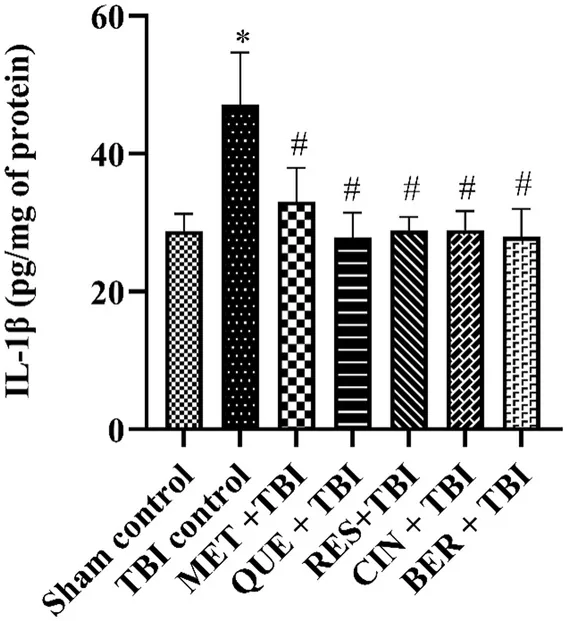
Pretreatment effects with AMPK (adenosine monophosphate–activated protein kinase) activators on IL‐1β (interleukin‐1β) levels were measured using ELISA (enzyme‐linked immunosorbent assay). Data are shown as mean ± SEM (standard error of the mean; *N* = 4); one‐way ANOVA (analysis of variance) and Tukey's post hoc test. *p < 0.05 versus sham control and ^#^ *p* < 0.05 versus TBI (traumatic brain injury) rats. BER, berberine; CIN, cinnamaldehyde; MET, metformin; QUE, quercetin; RES, resveratrol.

Elevated IL‐1β levels were observed in the TBI control group compared with sham control. (*p* < 0.05) (Figure 10). Pretreatment with MET significantly lowered the elevated IL‐1β levels compared to TBI rats (*p* < 0.05). Similarly, pretreatment with AMPK activators showed a significant reduction in TBI‐induced IL‐1β levels (*p* < 0.05) (Table 8; Figure 10). These results indicate that pretreatment with AMPK activators may have anti‐inflammatory effects by mitigating NLRP3 inflammasome–mediated IL‐1β activation after weight‐drop‐induced TBI.

**TABLE 8:** Effects of pretreatment with AMPK activators on IL‐1β levels in TBI‐induced neuroinflammation in rats.

Serial number	Treatment	IL‐1β levels (pg/mg) (mean ± SEM)
1	Sham control	28.79 ± 1.2
2	TBI control	47.11 ± 3.7*
3	Metformin + TBI	33.09 ± 2.4^#^
4	Quercetin + TBI	27.86 ± 1.7^#^
5	Resveratrol + TBI	28.85 ± 0.9^#^
6	Cinnamaldehyde + TBI	28.94 ± 1.3^#^
7	Berberine + TBI	27.98 ± 1.9^#^

Abbreviations: AMPK, adenosine monophosphate–activated protein kinase; IL‐1β, interleukin‐1β; SEM, standard error of the mean; TBI, traumatic brain injury.

* *p* < 0.05 versus sham control.

^#^ *p* < 0.05 versus TBI rats.

### 3.8 Effects of pretreatment with AMPK activators on CD86 and CD206 polarization marker levels in TBI‐induced neuroinflammation in rats

**FIGURE 11:**
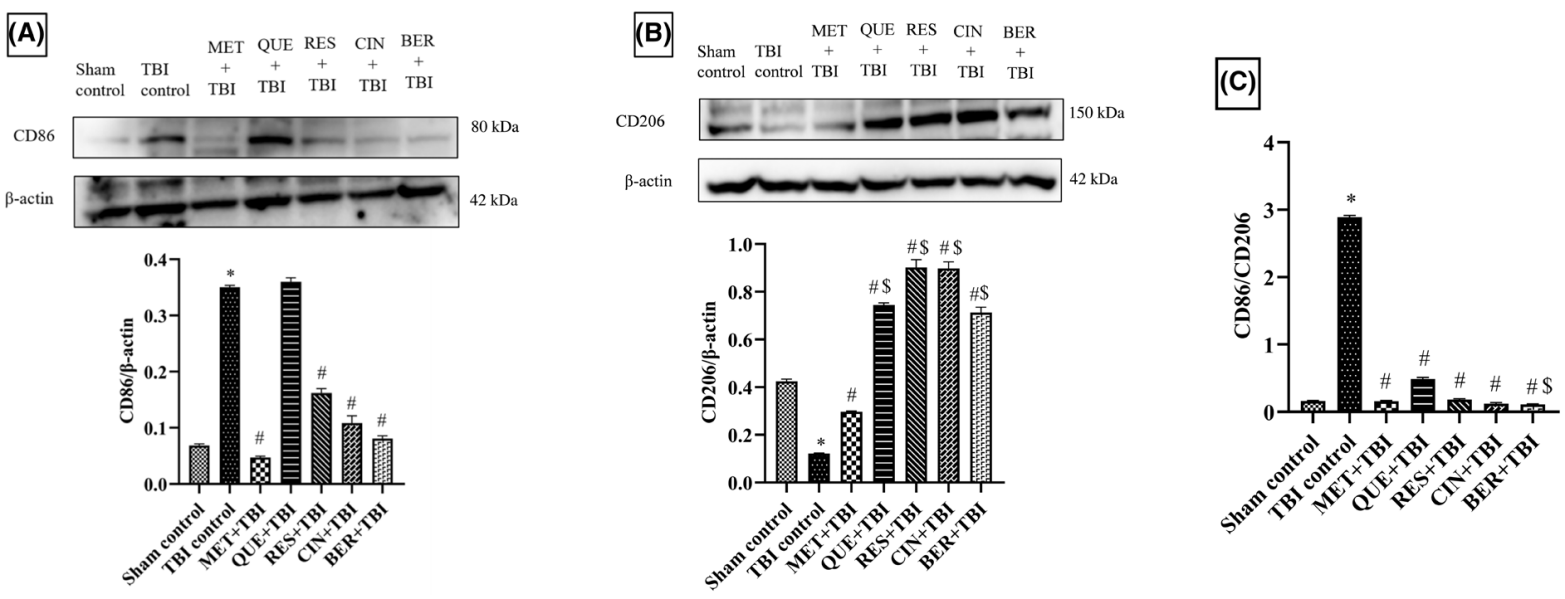
(A) Effects of pretreatment with AMPK (adenosine monophosphate–activated protein kinase) activators on CD86 in brain homogenates from TBI (traumatic brain injury)–induced neuroinflammation in rats were analyzed using Western blot, with β‐actin used as normalization; mean ± SEM (standard error of the mean; *N* = 3); one‐way ANOVA (analysis of variance) and Tukey's post hoc test. (B) Effects of pretreatment with AMPK (adenosine monophosphate–activated protein kinase) activators on CD206 in brain homogenates from TBI (traumatic brain injury)–induced neuroinflammation in rats were analyzed using Western blot, with β‐actin used as normalization; one‐way ANOVA (analysis of variance) and Tukey's post hoc test; mean ± SEM (standard error of the mean; *N* = 3). (C) Effects of pretreatment with AMPK (adenosine monophosphate–activated protein kinase) activators on CD86/CD206 ratio in brain homogenates from TBI (traumatic brain injury)–induced neuroinflammation in rats were analyzed using Western blot, with β‐actin used as normalization; mean ± SEM (standard error of the mean; *N* = 3); one‐way ANOVA (analysis of variance) and Tukey's post hoc test. *p < 0.05 versus sham control, ^#^ *p* < 0.05 versus TBI rats, and ^$^ *p* < 0.05 versus metformin pretreatment in TBI rats. BER, berberine; CIN, cinnamaldehyde; MET, metformin; QUE, quercetin; RES, resveratrol.

An increase in CD86 levels in TBI rats was observed compared to sham control (*p* < 0.05), indicating the pro‐inflammatory state with the weight drop, reversed by pretreatment with MET (*p* < 0.05) (Figure 11A). Similarly, it was found that RES, CIN, and BER pretreatment significantly decreased the TBI‐induced CD86 levels (*p* < 0.05) (Table 9). However, QUE pretreatment did not show any effect on TBI‐induced CD86 levels.

**TABLE 9:** Effects of pretreatment with AMPK activators on CD86 and CD206 polarization marker levels in TBI‐induced neuroinflammation in rats.

Serial number	Treatment	CD86/β‐actin levels (mean ± SEM)	CD206/β‐actin levels (mean ± SEM)	CD86/CD206 ratio (mean ± SEM)
1	Sham control	0.07 ± 0.002	0.42 ± 0.006	0.16 ± 0.005
2	TBI control	0.35 ± 0.002*	0.12 ± 0.001*	2.89 ± 0.02*
3	Metformin + TBI	0.05 ± 0.001^#^	0.3 ± 0.001^#^	0.16 ± 0.004^#^
4	Quercetin + TBI	0.36 ± 0.004	0.74 ± 0.005^#^, ^$^	0.48 ± 0.01^#^
5	Resveratrol + TBI	0.16 ± 0.004^#^	0.9 ± 0.01^#^, ^$^	0.18 ± 0.01^#^
6	Cinnamaldehyde + TBI	0.11 ± 0.008^#^	0.9 ± 0.01^#^, ^$^	0.12 ± 0.01^#^
7	Berberine + TBI	0.08 ± 0.003^#^	0.71 ± 0.01^#^, ^$^	0.11 ± 0.002^#^, ^$^

Abbreviations: AMPK, adenosine monophosphate–activated protein kinase; SEM, standard error of the mean; TBI, traumatic brain injury.

* *p* < 0.05 versus sham control.

^#^ *p* < 0.05 versus TBI rats.

^$^ *p* < 0.05 versus metformin pretreatment in TBI rats.

In TBI rats, there is a significant reduction in CD206 levels compared with sham control (*p* < 0.05) (Figure 11B). MET pretreatment significantly enhanced the decline in CD206 levels compared with TBI rats (*p* < 0.05). Similarly, pretreatment with AMPK activators also significantly increased the decreased levels of CD206 compared with TBI rats (*p* < 0.05). Compared to the MET pretreatment group, all the AMPK activators significantly increased the CD206 levels (*p* < 0.05) (Figure 11B; Table 9), depicting an anti‐inflammatory state.

There is a shift from pro‐inflammatory (CD86) to anti‐inflammatory (CD206) polarization markers in brain tissue upon pretreatment with AMPK‐activating compounds, as evidenced by a decreased CD86/CD206 ratio. BER pretreatment reduced the CD86/CD206 ratio compared with MET pretreatment (*p* < 0.05) (Figure 11C).

### 3.9 Effects of pretreatment with AMPK activators on TNF‐α levels in TBI‐induced neuroinflammation in rats

**FIGURE 12:**
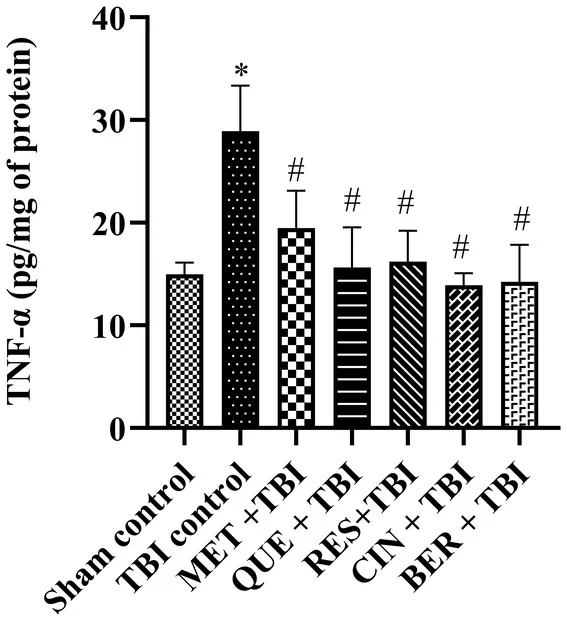
Effects of pretreatment with AMPK (adenosine monophosphate–activated protein kinase) activators on secondary inflammatory mediators like TNF‐α (tumor necrosis factor‐α) in brain homogenates from TBI (traumatic brain injury)–induced neuroinflammation in rats were measured using ELISA (enzyme‐linked immunosorbent assay); mean ± SEM (standard error of the mean; *N* = 4); one‐way ANOVA (analysis of variance) and Tukey's post hoc test. *p < 0.05 versus sham control and ^#^ *p* < 0.05 versus TBI rats. BER, berberine; CIN, cinnamaldehyde; MET, metformin; QUE, quercetin; RES, resveratrol.

TBI rats exhibited significantly higher TNF‐α levels than sham controls (*p* < 0.05), as shown in Figure 12. Pretreatment with MET significantly reduced the higher TNF‐α levels compared with TBI rats (*p* < 0.05). Pretreatment with AMPK activators significantly reduced the TBI‐induced TNF‐α levels (*p* < 0.05) (Table 10).

**TABLE 10:** Effects of pretreatment with AMPK activators on secondary inflammatory mediators like TNF‐α in TBI‐induced neuroinflammation in rats.

Serial number	Treatment	TNF‐α levels (pg/mg) (mean ± SEM)
1	Sham control	14.99 ± 0.5
2	TBI control	28.91 ± 2.2*
3	Metformin + TBI	19.48 ± 1.8^#^
4	Quercetin + TBI	15.65 ± 1.9^#^
5	Resveratrol + TBI	16.23 ± 1.4^#^
6	Cinnamaldehyde + TBI	13.91 ± 0.5^#^
7	Berberine + TBI	14.24 ± 1.8^#^

Abbreviations: AMPK, adenosine monophosphate–activated protein kinase; SEM, standard error of the mean; TBI, traumatic brain injury; TNF‐α, tumor necrosis factor‐α.

* *p* < 0.05 versus sham control.

^#^ *p* < 0.05 versus TBI rats.

### 3.10 Effects of pretreatment with AMPK activators on brain weight in TBI‐induced neuroinflammation in rats

**FIGURE 13:**
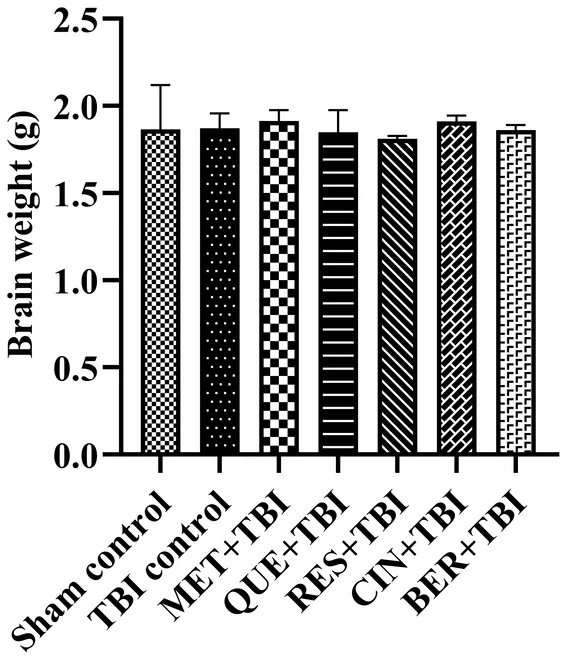
Effect of pretreatment with AMPK (adenosine monophosphate–activated protein kinase) activators on brain weight from TBI (traumatic brain injury)–induced neuroinflammation in rats. Data are shown as mean ± SEM (standard error of the mean; *N* = 4–6); one‐way ANOVA (analysis of variance) and Tukey's test. BER, berberine; CIN, cinnamaldehyde; MET, metformin; QUE, quercetin; RES, resveratrol.

No statistically significant change was observed in the brain weights of any of the groups, as shown in Figure 13 (Table 11).

**TABLE 11:** Effect of pretreatment with AMPK activators on brain weight in TBI‐induced neuroinflammation in rats.

Serial number	Treatment	Brain weight (mean ± SEM)
1	Sham control	1.87 ± 0.1
2	TBI control	1.87 ± 0.04
3	Metformin + TBI	1.91 ± 0.03
4	Quercetin + TBI	1.85± 0.05
5	Resveratrol + TBI	1.81± 0.01
6	Cinnamaldehyde + TBI	1.91 ± 0.02
7	Berberine + TBI	1.86 ± 0.01

Abbreviations: AMPK, adenosine monophosphate–activated protein kinase; SEM, standard error of the mean; TBI, traumatic brain injury.

### 3.11 Effects of pretreatment with AMPK activators on TBI‐induced histopathological changes in cerebrum and hippocampus brain regions

**FIGURE 14:**
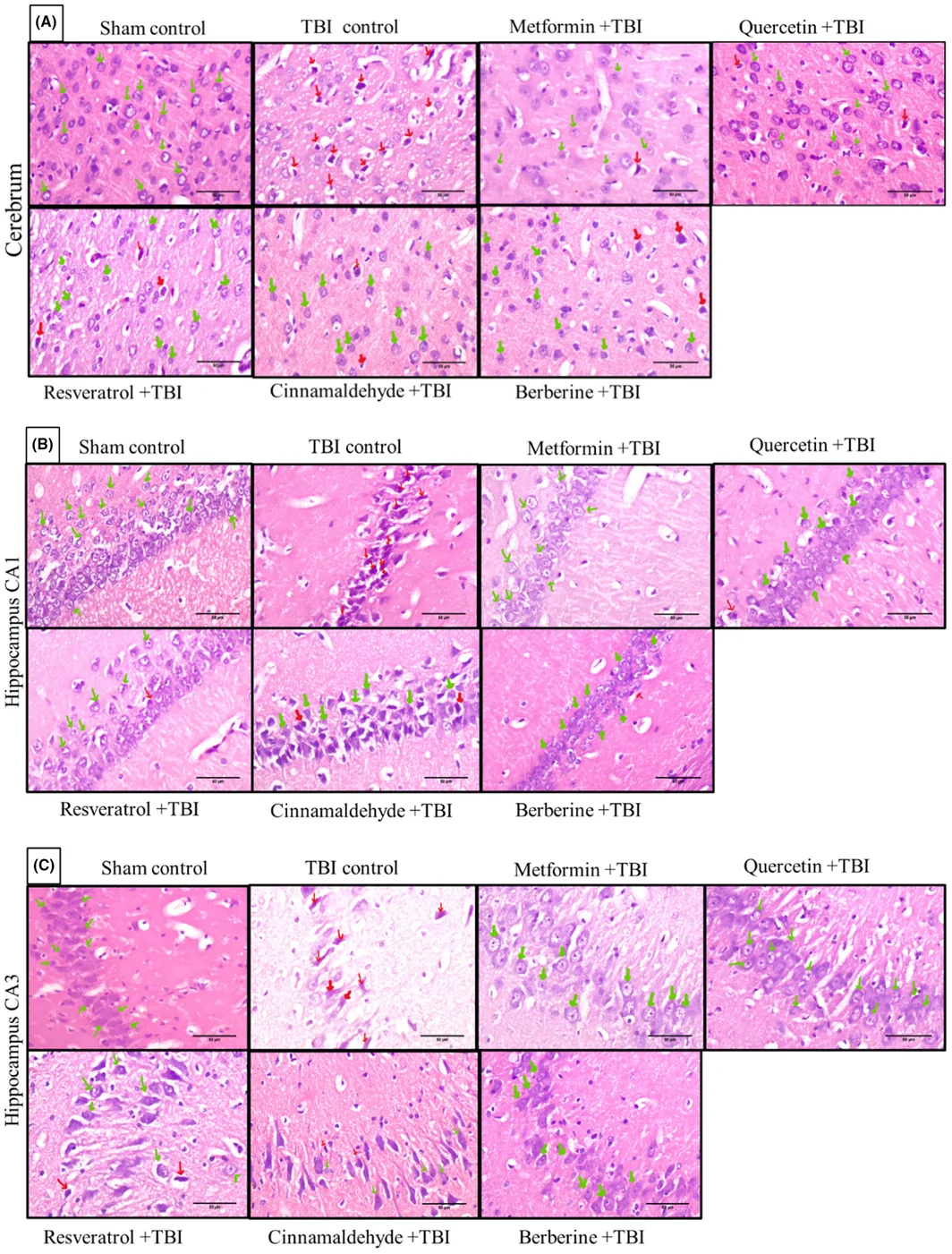
H&E (hematoxylin and eosin) staining was performed on rat brain sections (*N* = 2) from the TBI (traumatic brain injury)‐induced neuroinflammation model, focusing on the following regions: (A) cerebrum, (B) CA1 region of the hippocampus, and (C) CA3 region of the hippocampus. The red arrows indicate degenerated neurons, and the green arrows indicate healthy neurons. All the images were captured at 400× magnification; scale bar: 50 μm. BER, berberine; CIN, cinnamaldehyde; MET, metformin; QUE, quercetin; RES, resveratrol.

In TBI rats, several regions in the cerebrum and hippocampus exhibited degeneration, appearing dark and basophilic compared with the sham control group. In the sham control group, the neurons appeared healthy, characterized by pale, round nuclei with well‐defined nuclear boundaries and prominent nucleoli (Figure 14A–C). Pretreatment with AMPK activators effectively reduced the number of degenerated neurons and mitigated TBI‐induced histological changes in the cerebrum and hippocampus. These findings highlight that AMPK activators could exert neuroprotective effects in mitigating neuronal damage caused by weight‐drop‐induced TBI.

## 4 DISCUSSION

This study employed a rat model of TBI using the weight‐drop model that produces cognitive deficits, as well as cellular, molecular, and histological changes resembling those in human TBI.^21^ This weight‐drop model is a widely used method to simulate TBI in rodents.^12^ This study reported that cognitive decline, oxidative stress, neuroinflammation, and histological changes in rats after TBI were mitigated by pretreatment with AMPK activators, indicating the neuroprotective effect of AMPK‐activating compounds.

The pathophysiology of TBI is complex, involving (a) primary and (b) secondary mechanisms. The primary injury results from direct physical impact, leading to neuronal cell death and axonal damage, whereas secondary processes, including oxidative stress, DNA damage, mitochondrial dysfunction, neuroinflammation, and other pathological changes, occur. These secondary events further disrupt cellular homeostasis and contribute to ongoing degeneration and BBB disruption. Collectively, these mechanisms underlie the persistent neurological and cognitive symptoms often observed even after brain trauma.^22^, ^23^, ^24^ There is strong evidence indicating that TBI, especially of moderate to severe severity, is a crucial factor for developing neurodegenerative diseases, like Alzheimer's disease (AD) and Parkinson's disease (PD).^25^, ^26^

The cortex and hippocampus are two brain regions commonly affected by head trauma. Because the hippocampal neurons are crucial for learning and memory, their selective vulnerability contributes to frequent memory impairment.^27^, ^28^ Our experimental study revealed that TBI rats failed to distinguish between familiar and novel objects, as evidenced by the lower RI and DI on days 8 and 16. These are in line with the prior reports showing impairment in recognition memory in rodents and humans after TBI.^24^, ^29^, ^30^ We further evaluated the effects of pretreatment with AMPK activators on TBI‐induced decline in visual recognition memory on days 8 and 16. Our treatment protected against these short‐ and long‐term visual recognition memory impairments, as indicated by the enhanced RI and DI. These findings are supported by previous literature, where MET,^31^ QUE,^32^, ^33^ and RES^34^ significantly restored head trauma‐induced recognition memory by spending more time with the novel object on days 8 and 16. However, CIN and BER did not exhibit a significant effect on RI on day 8, as confirmed by previous reports.^35^, ^36^ The discrepancy between RI and DI is not unexpected because these indices are calculated using different formulas and therefore differ in their sensitivity to changes in exploratory behavior. The RI reflects the proportion of total exploration time devoted to the novel object, whereas the DI reflects the relative preference for the novel object over the familiar object. Consequently, a treatment may significantly improve the DI without producing a statistically significant change in the RI.

No significant change in visual recognition memory was observed between days 8 and 16, except for BER. A possible reason could be that cognitive function (like recognition memory) after TBI may plateau temporarily, staying at the same level without significant change during that short interval. Future long‐term studies should explore more sensitive tasks and extended follow‐up to detect delayed improvements. Additionally, memory improvement may depend on long‐term structural repair, which might not be evident within an 8‐day gap.

Memory impairment is a common outcome of head trauma, affecting individuals across all age groups, with a significant impact on the young population. Studies conducted on rodents reported that head injury can result in spatial learning and memory deficits.^37^, ^38^, ^39^, ^40^ Similarly, our study demonstrated that TBI‐challenged rats exhibited significant spatial learning and memory impairments, likely due to hippocampal damage observed in histopathology studies. This was evidenced by increased escape latency, distance traveled, decreased island entries, and path efficiency in MWM.

Pretreatment with AMPK activators significantly ameliorated the memory deficits associated with TBI, as indicated by reduced escape latency, number of island entries, and distance traveled. These findings align with earlier studies showing that MET enhances long‐term spatial memory retention by AMPK activation, suggesting that AMPK phosphorylation could benefit TBI. Activation of AMPK has also been associated with enhanced expression of cytoprotective proteins such as nuclear factor erythroid 2‐related factor 2 (Nrf2) and heme oxygenase‐1 (HO‐1),^11^, ^41^, ^42^ suggesting that the observed memory improvement and neuroprotection may result from these AMPK‐mediated mechanisms.

In addition to MET, several natural compounds have shown beneficial effects on cognitive outcomes after TBI. QUE improved cognitive outcome by mitigating neuronal autophagy and apoptosis through the Phosphoinositide 3‐kinase(PI3K)/Akt pathway activation.^43^, ^44^ RES attenuated cognitive deficits by suppressing oxidative stress–mediated apoptosis and activating p38 signaling.^45^, ^46^ BER also restored spatial learning and memory through the regulation of Sirt1/p38 mitogen‐activated protein kinase (MAPK) expression in the mouse TBI model.^47^, ^48^ Similarly, CIN was found to mitigate cognitive impairments in the rat model of TBI.^35^ There is no significant change in the treatment groups across the number of entries to the target quadrant and time spent in the target quadrant in MWM.

The hippocampus is highly vulnerable to oxidative stress, contributing to secondary damage in the ipsilateral CA1 region.^48^ Oxidative stress is a well‐established factor in the development of TBI, resulting from an imbalance between oxidants and antioxidants, which causes excessive production of reactive oxygen and nitrogen species. These free radicals damage cellular components, impair mitochondria, and cause neuronal death. Secondary brain injury involves the production of immune mediators and the onset of acute oxidative stress.^49^, ^50^ Involvement of oxidative damage markers in rat models of TBI further highlights the involvement of oxidative stress in the progression of injury.^51^, ^52^, ^53^ Similarly, our experimental study enhanced lipid peroxidation in the brain (as observed by high MDA levels), indicative of secondary inflammation, which may be due to enhanced inflammatory mediators, in rats subjected to TBI.

Furthermore, pretreatment with AMPK activators alleviated the TBI‐induced MDA levels in the brain. This observation was consistent with prior studies showing that MET,^15^ QUE,^2^ RES,^54^ CIN,^55^ and BER^47^ mitigated lipid peroxidation by reducing MDA levels after TBI. Possible reasons could be that AMPK is a crucial regulator of inflammation and reactive oxygen species (ROS) production, and its interaction with Nrf2 highlights a novel link between oxidative stress and energy homeostasis. Its activation after TBI enhances antioxidant defense by promoting free radical scavenging and enzyme production.^56^

After TBI, a significant reduction in GSH levels is commonly observed in the brain, reflecting enhanced oxidative stress and secondary inflammation. A study reported a time‐dependent decline in hippocampal GSH levels in rats with moderate TBI, with significant decreases starting at 3 h and peaking between 24 and 48 h postinjury. Similarly, clinical data show reduced CSF‐GSH levels in infants and children with severe TBI from days 1 to 7 compared to healthy controls.^57^, ^58^ Similar to prior studies, TBI led to decreased GSH levels in the present study, indicating oxidative stress involvement. Moreover, pretreatment with MET, QUE, and BER alleviated TBI‐induced oxidative stress by enhancing the GSH levels in the brain.^2^ However, the limited effect of RES and CIN on GSH levels may be due to the short treatment duration, as GSH restoration likely requires sustained activation of antioxidant pathways.

Histopathological changes in the cerebrum and hippocampus were evaluated using H&E staining to better understand the causes of cognitive deficits and oxidative stress after TBI. Our experimental study demonstrated that TBI rats exhibited degenerative neurons with histopathological changes in the cerebrum and hippocampal areas, reflecting the cognitive impairments and oxidative stress. Moreover, pretreatment with AMPK activators reduced the degenerative neurons induced by TBI. These results are consistent with prior literature.^33^, ^59^, ^60^ To explore the neuroprotective role of AMPK activation in TBI, we assessed the p‐AMPK/AMPK ratio in the brain using Western blot. The literature suggests that AMPK activation could mitigate TBI‐induced neuronal damage. Loss or inhibition of AMPK after TBI results in increased inflammatory cytokine production, oxidative stress, and worsened outcomes by activating inflammasomes. AMPK activation restores energy balance, suppresses detrimental inflammation, and may improve cognitive outcomes, suggesting that enhancing AMPK activity mitigates the secondary effects of TBI.^11^, ^61^ As expected, pretreatment with RES, CIN, and BER significantly enhanced the decreased p‐AMPK/AMPK in the brain in TBI‐subjected rats. However, MET and QUE apparently enhanced the p‐AMPK/AMPK ratio, but the difference was statistically insignificant. This might be the restricted penetration of the BBB, and lower doses may fail to activate AMPK. A study showed that a MET dose of 200 mg/kg enhanced the p‐AMPK/AMPK ratio more effectively than the 100‐mg/kg dose and alleviated neurological deficits in TBI‐subjected rats.^1^

Neuroinflammation is a key pathological feature of secondary injury after TBI, with NF‐κB and the NLRP3 inflammasome playing central roles in mediating this response.^62^, ^63^ In TBI, NF‐κB is activated and upregulates NLRP3, whereas a secondary signal (such as ionic flux, mitochondrial dysfunction, or ROS) triggers NLRP3 inflammasome assembly. This results in the activation of caspase‐1, which helps produce IL‐1β and IL‐18, and increases other inflammatory markers like TNF‐α,^4^, ^5^, ^10^, ^64^ highlighting its involvement in inflammation.

NLRP3 is detected in neurons, astrocytes, and microglia of the cortex. Increased NLRP3 levels during brain swelling after TBI may be key in triggering inflammation. NLRP3 mRNA levels peaked at 6 h, decreased at 24 h, and then gradually increased up to 7 days, whereas its protein levels exhibited a significant time‐dependent increase from 24 h to 7 days postinjury.^10^ Both protein and mRNA levels of caspase‐1 increased in a time‐dependent manner from 6 h to 7 days postinjury in rodents with TBI^10^ and in patients.^65^, ^66^ IL‐1β is an inflammatory cytokine produced after NLRP3 inflammasome activation. Evidence suggests that both IL‐1β and IL‐18 play key roles in initiating and promoting the inflammatory cascade after TBI.^64^, ^67^ In both human and rodent models, increased IL‐1β levels have been observed in the CSF and brain parenchyma shortly after brain injury.^68^ IL‐1β concentrations in the tissue surrounding the injury site increased rapidly at 6 h postinjury and began to decline by 24 h.^10^ Elevated IL‐1β levels indicate NLRP3 inflammasome activation after TBI. Consistent with the earlier reports, this study demonstrates that the NLRP3/pro‐caspase‐1/IL‐1β pathway was activated in the brain in TBI‐subjected rats, along with the enhanced NF‐κB phosphorylation, which results in neuronal damage.

AMPK activation is essential for neuroprotection after TBI by suppressing the activation of NLRP3 inflammasome. AMPK signaling, through pathways involving Sirt1, FoxO1, and NF‐κB, contributes to reduced inflammation, oxidative stress, and neuronal apoptosis.^69^, ^70^ Additionally, AMPK activation has been linked to enhanced autophagy, which further supports the suppression of NLRP3 inflammasome activation.^71^ Furthermore, enhanced AMPK activity alleviated the TBI‐associated neuronal damage by suppressing NLRP3 inflammasome.^61^

In this study, pretreatment with AMPK activators reduced neuroinflammation by suppressing the p‐NF‐kB and NLRP3/pro‐caspase‐1/IL‐1β pathway in the brain. These findings are corroborated by published studies, which demonstrated that MET improves neurobehavioral outcomes after TBI by activating AMPK, regulating NF‐κB, modulating pro‐inflammatory to anti‐inflammatory polarization, and inhibiting NLRP3 inflammasome activation.^72^, ^73^ RES mitigates the inflammatory response after TBI by mitigating NLRP3 inflammasome activation via Sirt1.^74^ BER also reduced neuronal death and apoptosis by suppressing TLR4/MyD88/NF‐kB signaling in TBI.^36^, ^75^

Microglia serve as the brain's first line of defense after a mild injury, playing a central role in neuroinflammation after TBI. However, in severe brain injury, microglia become dysregulated by releasing cytotoxic mediators that hinder central nervous system (CNS) recovery and promote neuronal dysfunction and apoptosis.^76^ Microglial activation after TBI is a hallmark of neuroinflammation. Within 24 h after injury, microglia and astrocytes infiltrate the brain parenchyma due to BBB disruption. Activated microglia release large amounts of the pro‐inflammatory markers.^37^ In this study, TBI rats enhanced the pro‐inflammatory CD86 and decreased the anti‐inflammatory CD206 polarization markers in the brain, indicating the presence of inflammation after TBI. These findings align with the previous studies showing enhanced CD86 production within the cerebral cortex on the seventh day and reduced CD206 after TBI.^77^, ^78^

In this study, Western blot data revealed that pretreatment with AMPK activators significantly reduced CD86 production, except for QUE, and significantly enhanced CD206 levels in TBI rat brain, suggesting anti‐inflammatory activity, which is likely to underlie the cognitive improvement in TBI rats. The possible reason for this observation could be that AMPK activators enhance the polarization shifts from pro‐inflammatory to anti‐inflammatory via AMPK, helping to reduce the neurotoxic effects and alleviating secondary injury processes after TBI. There is no existing evidence showing that AMPK activators can inhibit the NLRP3 inflammasome, promote polarization shift, and reduce neuroinflammation through AMPK signaling in TBI models. These findings are reported experimentally for the first time.

Secondary neuroinflammatory processes occurring after microglial activation in TBI exposure were evaluated by performing ELISA for TNF‐α levels. Neuroinflammation after TBI is extensively studied, and it is evident that the brain significantly influences the inflammatory response after injury.^79^, ^80^ High levels of TNF‐α increase glutamate toxicity and BBB leakage. TNF‐α is harmful to neurons and can cause cell death through both necrosis and apoptosis.^81^

TBI caused a significant increase in TNF‐α levels in both the cortex and hippocampus and led to a neuroinflammatory cascade in rodent studies.^37^, ^38^ Similarly, this study reported an increase in inflammatory marker TNF‐α levels in TBI rat brains that significantly reduced after pretreatment with AMPK‐activating compounds, which supports the neuroprotective effect by mitigating secondary neuronal damage and neurological dysfunction. Our findings are in line with studies showing that MET reduced TNF‐α levels associated with neurological deficits in TBI.^72^ Similarly, QUE alleviates microglial‐induced TNF‐α levels via the Peroxisome proliferator‐activated receptor gamma coactivator 1‐alpha (PGC‐1α)/Nrf2 pathway^82^; RES decreases hippocampal TNF‐α expression at 12, 24, and 48 h postinjury^83^; and CIN exerts neuroprotective effects by alleviating TNF‐α in TBI.^35^ There is no change in brain weight in any of the groups.

Further studies using a cleavage‐specific caspase‐1 antibody are warranted to directly assess caspase‐1 activation and provide more definitive evidence of NLRP3 inflammasome activation. Also, incorporating compound C, MCC950, or complementary genetic approaches (e.g., AMPK or NLRP3 knockdown/knockout models) will be necessary to establish the direct mechanistic relationship between AMPK activation and NLRP3 inflammasome inhibition in TBI. Additionally, the present study employed a pretreatment strategy, which limits its direct clinical translation because patients receive treatment only after TBI. Future studies should investigate the efficacy of AMPK activators when administered after injury at clinically relevant time points to determine the therapeutic window and validate their translational potential.

## 5 CONCLUSION

This study establishes a relationship between AMPK activation and NLRP3 inflammasome inhibition in TBI using the weight‐drop method in rats. Our findings demonstrate that pretreatment with AMPK activators, including MET, QUE, RES, CIN, and BER, alleviates TBI‐induced neuroinflammation in rats. These compounds improved visual recognition and spatial memory, as further supported by histopathological studies. The observed neuroprotection is likely mediated through AMPK activation, which suppressed NF‐κB and NLRP3 inflammasome signaling. Compared to MET, CIN, BER, and RES exhibited a significant effect on the distance‐traveled parameter of spatial memory and downregulated the NF‐κB/NLRP3 inflammasome pathway.

Moreover, pretreatment with AMPK activators promoted a shift from pro‐inflammatory to anti‐inflammatory effects and reduced secondary inflammation. Compared with MET, BER exhibited a significant effect (CD86/CD206 ratio). The model we describe could act as a helpful research tool to determine the mechanistic foundations of TBI. This model provides a relevant platform for evaluating potential therapeutic interventions targeting neuroinflammation and cognitive deficits associated with TBI.

## AUTHOR CONTRIBUTIONS

**Anoop Kishore:** Conceptualization; methodology; investigation; validation; writing – review and editing; supervision; formal analysis. **Triveni Kodi:** Conceptualization; methodology; data curation; validation; writing – original draft; investigation; formal analysis. **Krishnadas Nandakumar:** Writing – review and editing; supervision; investigation. **Paka Sravan Kumar:** Methodology; investigation.

## FUNDING INFORMATION

This work was supported by the Senior Research Fellowship (SRF) grant from the Indian Council of Medical Research (ICMR‐SRF) to Triveni Kodi (3/1/2/149/Neuro/2021‐NCD‐I) and the Intra‐Mural Fund (IMF) provided by Manipal Academy of Higher Education, Manipal, India, for conducting the research.

## CONFLICT OF INTEREST STATEMENT

The authors declare no conflict of interest.

## ETHICS STATEMENT

The study was approved by the IAEC (no. IAEC/KMC/100/2022), Kasturba Medical College (KMC), Manipal Academy of Higher Education (MAHE), Manipal, India. All animal experiments were conducted in accordance with the guidelines established for the use and care of laboratory animals by the Committee for Control and Supervision of Experiments on Animals (CCSEA), Government of India.

## Data Availability

The datasets and materials used and/or analyzed for the current study are available upon reasonable request.
